# SUMOylation mediates CtIP’s functions in DNA end resection and replication fork protection

**DOI:** 10.1093/nar/gkaa1232

**Published:** 2021-01-06

**Authors:** Andrew J Locke, Lazina Hossain, Glynnis McCrostie, Daryl A Ronato, Amira Fitieh, Tanzeem Ahmed Rafique, Fatemeh Mashayekhi, Mobina Motamedi, Jean-Yves Masson, Ismail Hassan Ismail

**Affiliations:** Division of Experimental Oncology, Department of Oncology, Faculty of Medicine & Dentistry, University of Alberta; Cross Cancer Institute, Edmonton, Alberta, T6G 1Z2, Canada; Division of Experimental Oncology, Department of Oncology, Faculty of Medicine & Dentistry, University of Alberta; Cross Cancer Institute, Edmonton, Alberta, T6G 1Z2, Canada; Division of Experimental Oncology, Department of Oncology, Faculty of Medicine & Dentistry, University of Alberta; Cross Cancer Institute, Edmonton, Alberta, T6G 1Z2, Canada; Oncology Division, CHU de Québec-Université Laval Research Center, Québec City, Québec, G1R 3S3, Canada; Department of Molecular Biology, Medical Biochemistry and Pathology, Faculty of Medicine; Laval University Cancer Research Center, Université Laval, Québec City, Québec, G1V 0A6, Canada; Division of Experimental Oncology, Department of Oncology, Faculty of Medicine & Dentistry, University of Alberta; Cross Cancer Institute, Edmonton, Alberta, T6G 1Z2, Canada; Biophysics Department, Faculty of Science, Cairo University, Giza, Egypt; Division of Experimental Oncology, Department of Oncology, Faculty of Medicine & Dentistry, University of Alberta; Cross Cancer Institute, Edmonton, Alberta, T6G 1Z2, Canada; Division of Experimental Oncology, Department of Oncology, Faculty of Medicine & Dentistry, University of Alberta; Cross Cancer Institute, Edmonton, Alberta, T6G 1Z2, Canada; Division of Experimental Oncology, Department of Oncology, Faculty of Medicine & Dentistry, University of Alberta; Cross Cancer Institute, Edmonton, Alberta, T6G 1Z2, Canada; Oncology Division, CHU de Québec-Université Laval Research Center, Québec City, Québec, G1R 3S3, Canada; Department of Molecular Biology, Medical Biochemistry and Pathology, Faculty of Medicine; Laval University Cancer Research Center, Université Laval, Québec City, Québec, G1V 0A6, Canada; Division of Experimental Oncology, Department of Oncology, Faculty of Medicine & Dentistry, University of Alberta; Cross Cancer Institute, Edmonton, Alberta, T6G 1Z2, Canada; Biophysics Department, Faculty of Science, Cairo University, Giza, Egypt

## Abstract

Double-strand breaks and stalled replication forks are a significant threat to genomic stability that can lead to chromosomal rearrangements or cell death. The protein CtIP promotes DNA end resection, an early step in homologous recombination repair, and has been found to protect perturbed forks from excessive nucleolytic degradation. However, it remains unknown how CtIP’s function in fork protection is regulated. Here, we show that CtIP recruitment to sites of DNA damage and replication stress is impaired upon global inhibition of SUMOylation. We demonstrate that CtIP is a target for modification by SUMO-2 and that this occurs constitutively during S phase. The modification is dependent on the activities of cyclin-dependent kinases and the PI-3-kinase-related kinase ATR on CtIP’s carboxyl-terminal region, an interaction with the replication factor PCNA, and the E3 SUMO ligase PIAS4. We also identify residue K578 as a key residue that contributes to CtIP SUMOylation. Functionally, a CtIP mutant where K578 is substituted with a non-SUMOylatable arginine residue is defective in promoting DNA end resection, homologous recombination, and in protecting stalled replication forks from excessive nucleolytic degradation. Our results shed further light on the tightly coordinated regulation of CtIP by SUMOylation in the maintenance of genome stability.

## INTRODUCTION

The accurate transmission of genetic information to progeny is essential for living organisms. In eukaryotic cells, two problems that arise include double-strand breaks (DSBs), toxic DNA lesions where the phosphodiester backbone is severed in both strands (1), and replication stress, conditions that slow or halt replication fork progression (2). Both must be resolved to maintain the integrity of the genome.

To repair DSBs, cells in S/G_2_ phase rely on homologous recombination (HR) (3). Here, 5′–3′ nucleolytic trimming away from the DSB, known as DNA end resection, yields 3′ single-stranded DNA (ssDNA) overhangs that are bound by RPA complexes (4). Short-range end resection is initiated by the nuclease activities of the MRN (MRE11–RAD50–NBS1) complex and stimulated by the MRN cofactor CtIP ((carboxy-terminal binding protein) interacting protein) (5–7), after which exonucleases DNA2 and EXO1 catalyze long-range resection up to hundreds of nucleotides away from the DSB in collaboration with BLM and WRN helicases (8). End resection can be negatively regulated by the activities of PARP-1 (9), HELB (10) and the Shieldin complex (11). Eventually, RPA coating the ssDNA overhangs is removed by PALB2-BRCA2 (12) to facilitate RAD51-mediated invasion and use of the sister chromatid as a template for error-free repair (3).

On the other hand, to deal with replication stress, the course of replication forks can be reversed by the assembly of a protective four-way junction, made possible by coordinated annealing of the newly synthesized strands and re-annealing of the template strands (13). Fork progression can later be restarted when replicative conditions are improved or downstream obstacles are repaired (13). Despite replication fork reversal being a well-known phenomenon (14,15), knowledge of its mechanisms is incomplete and is currently an area of active research (16). Intriguingly, HR proteins have been implicated in fork reversal and restart. RAD51 has been found to mediate fork reversal (17), while also, along with BRCA1 and BRCA2, to protect reversed forks from excessive degradation by the nuclease activities of MRE11 and DNA2 (18–23), both of which are end resection factors in HR (8).

Recent evidence demonstrates a role for the end resection factor CtIP at the replication fork, beyond its established role in promoting end resection for HR (24–28). For example, CtIP was found to be enriched at ongoing replication forks in a proteomic screen (24), and was also found to interact with PCNA (proliferating cell nuclear antigen) (25), a clamp protein that enhances the processivity of DNA polymerases in DNA replication (29). This interaction targeted CtIP to foci of active DNA replication (25). While disrupting the interaction suppressed proliferation, caused cell cycle arrest, and induced DNA damage and a checkpoint response (25), the function of the interaction remains unknown. More recently, CtIP was shown to protect nascent DNA in reversed forks from excessive DNA2-dependent degradation in response to replication stress, independent of its role in HR-related end resection (26). Still, it is not clear how CtIP’s fork protective function is regulated.

The activity of HR proteins is tightly controlled by various reversible post-translational modifications (PTMs) by chemical groups (e.g. phosphorylation) or proteins (e.g. ubiquitylation) (30), and CtIP is no exception. It is ubiquitylated by BRCA1 (31) and RNF138 (32), the latter of which targets it to DSB sites. CtIP is also phosphorylated by the DNA damage sensing kinases ATM (ataxia telangiectasia mutated) and ATR (ataxia telangiectasia and Rad3-related), and cyclin-dependent kinases (CDKs), the phosphorylations promoting its role in end resection and mediating its interaction with BRCA1 (33–38). Another modification, SUMOylation, describes the conjugation of members of the small ubiquitin-like modifier (SUMO) protein family onto target proteins. Analogous to ubiquitylation, a cascade of E1, E2 and E3 enzymes catalyzes the covalent linkage of SUMO isoforms onto protein substrates, and SUMO-specific proteases facilitate SUMO removal from these modified targets (39). While conjugation of SUMO proteins to other HR proteins has been shown to regulate protein stability, activity, nuclear trafficking and protein–protein interactions (40–45), the role of SUMOylation in CtIP function is just beginning to be uncovered (46), and so far SUMOylation has not been linked to CtIP’s function in replication fork protection. In this study, we demonstrate that CtIP is modified by SUMO-2 in S phase in an ATR- and CDK-dependent manner and is mediated by an interaction with PCNA. We further implicate PIAS4 as a SUMO E3 ligase for CtIP during S phase, and identify a key site for CtIP SUMOylation at residue K578. Functionally, we demonstrate K578 SUMOylation promotes CtIP’s function at halted replication forks in protecting nascent DNA from uncontrolled degradation, and in HR by stimulating DNA end resection. Our findings suggest SUMOylation is a mechanistic link between CtIP’s established interaction with PCNA to its ability to regulate fork stability during replication stress.

## MATERIALS AND METHODS

### Plasmids, siRNAs, site-directed mutagenesis, transfections

pCAGGS empty vector and pCAGGS-I-*Sce*I were gifts from Jeremy Stark (City of Hope Comprehensive Cancer Center). Plasmids encoding Gam1-WT (wildtype) or -L258A/L265A were gifts from Susanna Chiocca (European Institute of Oncology IRCCS, Milan). FLAG-CtIP and a series of internal deletion mutants (D1 to D6) (47) were kind gifts from Junjie Chen (University of Texas). pICE-HA-CtIP-siR-WT (Addgene plasmid #82030; http://n2t.net/addgene:82030; RRID:Addgene_82030) and pICE-HA-CtIP-siR-S664A-S679A-S745A (Addgene plasmid #82031; http://n2t.net/addgene:82031; RRID:Addgene_82031) (48) were gifts from Patrick Calsou. FLAG-hPIAS4 (Addgene plasmid #15208; http://n2t.net/addgene:15208; RRID:Addgene_15208) (49) was a gift from Ke Shuai. siRNA-resistant N-terminally GFP-tagged CtIP plasmids (GFP-CtIP) of wildtype (WT), -7KR, -K896R, -6KR, -T847A and -T847E were generous gifts from Pablo Huertas (University of Sevilla). RFP-PCNA was a gift from Michael Hendzel (University of Alberta). Custom siRNA duplexes were obtained from Sigma-Aldrich (Supplementary Table S1) and transfected 48 h prior using Lipofectamine RNAiMax Transfection Reagent (Invitrogen, Thermo-Fisher) according to the manufacturer's instructions. CtIP siRNA was transfected at 20–25 nM. If two transfections were required, 10 nM siCtIP was used for a second transfection 24 h after the first one. All other siRNAs were used at 20–50 nM. Mutagenesis was performed using the Quikchange II XL (Agilent) and Q5 (New England Biolabs) site-directed mutagenesis kits according to the manufacturers’ instructions, generating GFP-CtIP-T859A, -T859E, -Δ515–518, -K578R, -K578R-K896R, -7KR-R578K, -N181A, -N181A-K578R, -N289A-H290A and -N289A-H290A-K578R, as well as pICE-HA-CtIP-siR-K578R (Supplementary Table S2). Plasmids containing the desired mutations were verified by Sanger sequencing performed at The Applied Genomics Core (University of Alberta) (Supplementary Table S3). Unless indicated otherwise, DNA plasmids were transfected using Effectene Transfection Reagent (Qiagen) 16–24 h prior to cell harvest.

### Human cell lines and tissue culture

U-2 OS, HEK293, HeLa and HeLa His_10_-SUMO-2 were cultured at 37°C in a humidified atmosphere containing 5% CO_2_ in low glucose DMEM (Dulbecco's Modified Eagle's medium) supplemented with 10% fetal bovine serum (Gibco), 50 units/ml penicillin and 50 μg/ml streptomycin (Life Technologies). U-2 OS cells stably expressing the DR-GFP cassette were a gift from Jeremy Stark (City of Hope Comprehensive Cancer Center). U-2 OS cells stably expressing GFP-CtIP were a gift from Steve Jackson (University of Cambridge). U-2 OS cells stably expressing MRE11-GFP were a gift from Dorthe Helena Payne-Larsen (Danish Cancer Society Research Center). HeLa cells stably expressing 10x-histidine-tagged SUMO-2 from a pLV-CMV-His_10_-SUMO-2-IRES-GFP construct (HeLa His_10_-SUMO-2) (50) or not (parental HeLa) were gifts from Alfred C.O. Vertegaal (Leiden University). U-2 OS cells stably expressing GFP-tagged CtIP constructs were generated by transient lipofection of the constructs followed by selection in 850 μg/ml G418 (Thermo-Fisher). Cells were seeded to achieve 70–90% final confluency at the time of harvest.

### Pharmacological treatments

Unless indicated otherwise, all inhibitors were purchased from Millipore-Sigma or Selleck Chemicals. Inhibitors were diluted in warmed (37°C) tissue culture medium (low glucose DMEM supplemented with 10% fetal bovine serum), with working concentrations and treatment times indicated in the figure legends. For vehicle controls, the same dilutions were performed using the solvent of the inhibitor (either DMSO or water). As the activity of ginkgolic acid 15:1 (GA, Cayman Chemical) was inactivated in the presence of serum (data not shown), cells were rinsed twice in phosphate buffered saline (PBS) prior to treatment with GA to remove residual serum left from the culture medium. GA treatment solutions were prepared by diluting GA into serum-free warmed DMEM and added to near-confluent cell monolayers.

### *In vivo* gene conversion homologous recombination reporter assay

U-2 OS stably expressing the DR-GFP cassette (Supplementary Figure S1A) were transfected with I-*Sce*I using a Gene Pulser Xcell Electroporation System (Bio-RAD). Cells were harvested 24 h later, after which flow cytometry was performed using a BD FACSCanto II (BD Biosciences) to detect GFP^+^ cells upon gating for forward and side scatter.

### Pulsed-field gel electrophoresis assay

90% confluent U-2 OS cells grown on 6 cm dishes were treated as indicated and then trypsinized. 1 × 10^6^ cells per condition were resuspended into 25 μl of PBS, then embedded into 60 μl of molten 0.9% Low Melting Point Agarose (Invitrogen) in 0.5× TAE Buffer (20 mM Tris pH 8.5, 10 mM acetic acid, 0.5 mM EDTA). The mixture was cast into a mold on ice to form a gel plug of volume 80 μl. Each plug was then digested with agitation at 32°C in 0.5 ml of PFGE Lysis Buffer (100 mM EDTA, pH 8.0, 1% *N*-laurylsarcosine, 0.2% sodium deoxycholate, 1 mg/ml Proteinase K) for 48 h. The treated plugs were then washed three times 15 min each in TE Buffer (20 mM Tris–HCl pH 8.0, 50 mM EDTA) with gentle agitation at room temperature, then cut in half. The half-plugs were electrophoresed at 14°C, in duplicate, in a 0.9% Certified Megabase Agarose (Bio-RAD)/0.5× TAE gel containing 0.25 μg/ml ethidium bromide via the CHEF-DR III Variable Angle System (Bio-RAD). The running parameters were as follows: Block 1 (9 h, 120° included angle, 5.5 V/cm, 30–18 s switch time); Block 2 (6 h, 117° included angle, 4.5 V/cm, 18–9 s switch time); Block 3 (6 h, 112° included angle, 4.0 V/cm, 9–5 s switch time). Resolved gels were visualized on a Typhoon 9400 Variable Mode Imager (GE Healthcare), then quantified with ImageQuant 5.2 (GE Healthcare).

### *In vitro* SUMOylation assay

An *in vitro* SUMO-2 conjugation kit (K-715) was purchased from Boston Biochem. Reactions were assembled on ice using 2 μl of each component if needed (buffer, E1, E2, SUMO-2, Mg^2+^-ATP) and 140 ng of recombinant glutathione S-transferase (GST)-tagged human CtIP (Abnova). The volume was completed to 20 μl with laboratory-grade water. Reactions were allowed to proceed for 1 h at 37°C, then quenched by adding 6.7 μl of 4X SDS Sample Buffer, along with 2-mercaptoethanol to a concentration of 5%, then heating at 95°C for 5 min. The mixtures were resolved by SDS-PAGE and immunoblotted to detect the reaction components.

### Cell cycle synchronization

HeLa His_10_-SUMO-2 cells were synchronized to the G_1_/S transition by double thymidine block. In brief, the cells were seeded to 40–50% confluency and 4 mM deoxythymidine was added to the culture media for 14–18 h (first block). The cells were then released to progress through the cell cycle with two washes in PBS and cultured in warmed culture media for 8–12 h. 4 mM deoxythymidine was added to the culture media for another 14–18 h (second block). The cells were then released by two washes in PBS and replacing with warmed culture media for the duration needed for the required cell cycle phase (e.g. 3 h for mid-S phase, at least 11 h for G_1_ phase). If necessary, siRNA and DNA plasmid transfections were performed during release from the first thymidine block. U-2 OS cells were synchronized to S phase in the same manner, except they were released for 4 h after double thymidine block instead of 3 h.

### His pull-down of His_10_-SUMO-2 conjugates by Ni-NTA affinity purification

Samples were processed in a protocol adapted from Tatham and Hay (51). HeLa His_10_-SUMO-2 cells from two 10 cm dishes or one 15 cm dish were harvested by trypsinization and divided into three fractions: 5% was saved for cell cycle analysis by flow cytometry (Fraction C), 10% for input control (Fraction I), and 85% for His pull-down (Fraction H). Fraction C was resuspended into ice cold 70% ethanol in PBS and processed for DNA content analysis, whereas fractions I and H were pelleted and frozen in liquid nitrogen; all were stored at –80°C. Samples of Fraction H were resuspended into 10 ml of ice cold fresh Guanidine Lysis Buffer (6 M guanidine–HCl, 100 mM sodium phosphate buffer pH 8.0, 10 mM Tris, 5 mM imidazole, 5 mM 2-mercaptoethanol), sonicated for 1 min at amplitude 50 on a Model 705 Sonic Dismembrator fitted with a microtip probe (Fisher Scientific), and centrifuged at 1450g for 15 min. The lysate was then mixed with 200–300 μl of Ni-NTA agarose beads (Qiagen) pre-equilibrated in Guanidine Lysis Buffer and agitated overnight at 4°C. The beads were washed once in Guanidine Wash Buffer (6 M guanidine–HCl, 100 mM sodium phosphate buffer pH 8.0, 10 mM Tris, 0.1% Triton X-100, 10 mM imidazole, 5 mM 2-mercaptoethanol), once in Urea Wash Buffer A (8 M urea, 100 mM sodium phosphate buffer pH 8.0, 10 mM Tris, 0.1% Triton X-100, 10 mM imidazole, 5 mM 2-mercaptoethanol), and three times in Urea Wash Buffer B (8 M urea, 100 mM sodium phosphate buffer pH 6.3, 10 mM Tris, 0.1% Triton X-100, 5 mM 2-mercaptoethanol, pH 8.0); all buffers were prepared immediately before use. On occasion, the imidazole concentration in buffers was raised to 20 mM for increased stringency. The beads were then eluted with agitation in Ni-NTA Elution Buffer (150 mM Tris–HCl pH 6.7, 200 mM imidazole, 5% sodium dodecyl sulfate, 30% glycerol, 0.05% bromophenol blue, 5% 2-mercaptoethanol) for 30 min at 60°C. Samples of Fraction I were resuspended into 2× SDS-PAGE Sample Buffer (125 mM Tris pH 6.8, 4% sodium dodecyl sulfate, 20% glycerol, 0.1% bromophenol blue, 5% 2-mercaptoethanol), heated for 2 min at 95°C, and ultrasonicated in a Bioruptor (Diagenode) for 30 min (each cycle: 30 s on, 30 s off). For subsequent SDS-PAGE and immunoblotting, volumes representing ∼16–22-fold more of starting cellular content were loaded for Fraction H relative to Fraction I to enable suitable detection of the SUMOylated proteins, unless indicated otherwise.

### DNA content analysis for cell cycle profiling

Cells were fixed in 70% ethanol in 1× PBS overnight. They were then washed once in PBS, then treated with 100 μg/ml RNase A in PBS containing 3.8 mM sodium citrate for 30 min at 37°C with agitation. Propidium iodide was added to a final concentration of 50 μg/ml and incubated with the cells for 30 min. Flow cytometry was performed on the processed samples using a BD FACSCanto II (BD Biosciences) to detect propidium iodide fluorescence upon gating for forward and side scatter.

### Fractionation for chromatin enrichment

Cell pellets were resuspended into ice cold Extraction Buffer (25 mM HEPES pH 7.9, 300 mM sucrose, 50 mM NaCl, 1 mM EGTA, 3 mM MgCl_2_, 0.5% IGEPAL CA-630) supplemented with cOmplete protease inhibitor cocktail, phosSTOP phosphatase inhibitor cocktail (both from Roche), and 25 mM *N*-ethylmaleimide (NEM) and tumbled end-over-end for 20 min at 4°C. Samples were then centrifuged at 20 000g for 20 min at 4°C, after which the supernatant was recovered as the soluble fraction. The pellet, representing the chromatin-enriched fraction, was then resuspended into the buffer of choice and sonicated with a Model 705 Sonic Dismembrator fitted with a microtip probe (Fisher Scientific).

### Co-immunoprecipitation (Co-IP) assay

Cell pellets from 10 cm dishes were resuspended into 200 μl of ice-cold RIPA buffer (50 mM Tris–HCl pH 7.4, 150 mM NaCl, 1% IGEPAL CA-630, 0.25% sodium deoxycholate, 0.1% SDS, 1 mM EDTA) supplemented with 50 mM NEM, 2× cOmplete and 1× phosSTOP (both Roche) protease and phosphatase inhibitor cocktails. After agitation on ice for 30 min, samples were clarified by centrifugation at 20 000g at 4°C, upon which 10% of the supernatant was saved for the input control. The remainder of the supernatant was diluted in ice-cold Dilution Buffer (150 mM NaCl, 10 mM Tris–HCl pH 7.5, 0.5 mM EDTA) supplemented with 25 mM NEM, 1× cOmplete and 1× phosSTOP to a final volume 1.7 ml. This was mixed with 25 μl of Dilution Buffer-equilibrated GFP- or RFP-Trap agarose beads (Chromotek) and tumbled overnight. The beads were washed once in Dilution Buffer, then three times in Wash Buffer (500 mM NaCl, 10 mM Tris–HCl pH 7.5, 0.5 mM EDTA), before sample elution into 2× Sample Buffer (125 mM Tris–HCl pH 6.8, 4% sodium dodecyl sulfate, 20% glycerol, 0.1% bromophenol blue, 5% 2-mercaptoethanol) at 95°C for 10 min. Analysis of IP and input fractions was performed via SDS-PAGE and immunoblotting. For CoIP experiments involving GFP-CtIP-Δ515–518, RIPA buffer was replaced with NETMN buffer (500 mM NaCl, 1 mM EDTA, 50 mM Tris–HCl pH 7.4, 2.5 mM MgCl_2_, 0.5% IGEPAL CA-630) (25), which was then adjusted to a composition similar to Dilution Buffer prior to mixing with the beads.

### SDS-PAGE and immunoblotting

Unless indicated otherwise, cell lysates were prepared by resuspending cell pellets into 2× SDS-PAGE Sample Buffer (125 mM Tris–HCl pH 6.8, 4% sodium dodecyl sulfate, 20% glycerol, 0.1% bromophenol blue, 5% 2-mercaptoethanol), heating for 2 min at 95°C, and sonicating with a Model 705 Sonic Dismembrator fitted with a microtip probe (Fisher Scientific), generating whole cell extract. Lysates were electrophoresed in 5–15% polyacrylamide mini-gels handcast in 0.1% sodium dodecyl sulfate (SDS) and Tris buffer (pH 6.8 for stacking layer, pH 8.8 for resolving) in running buffer (25 mM Tris–HCl pH 8.3, 192 mM glycine, 0.1% SDS), then wet transferred onto nitrocellulose membrane in transfer buffer (25 mM Tris–HCl pH 8.3, 192 mM glycine, 20% methanol) for 6 h at 50 V or 90 min at 110 V. Total protein on membranes was visualized and quantified by staining with REVERT Total Protein Stain (LI-COR Biosciences). For immunoblot, membranes were blocked in 4% fish skin gelatin in Tris buffered saline (TBS) at room temperature and incubated in primary antibodies (Supplementary Table S4) diluted in TBS + 0.1% Tween-20 (TBST) overnight at 4°C or 1 h at room temperature. They were then washed in TBST, incubated for 1 h with secondary antibodies conjugated to horseradish peroxidase (HRP) or IRDye 680RD and 800CW (all LI-COR Biosciences) (Supplementary Table S5) in TBST at room temperature, and rinsed in TBST and TBS. Amersham ECL Prime Western Blotting Detection Reagent (GE Healthcare) was used to detect HRP according to the manufacturer's instructions. Immunoblots were acquired on the Odyssey Fc Imaging System (LI-COR Biosciences) and quantified by densitometry using Image Studio software (LI-COR Biosciences). When necessary, membranes were stripped in a buffer containing 1% SDS and 100 mM glycine, pH 2.2, for 1 h prior to re-blocking and re-probing with the appropriate primary and secondary antibodies.

### Immunofluorescence (IF) staining

U-2 OS cells were seeded onto #1$\frac{1}{2}$ cover slips (Electron Microscopy Sciences) at least 24 h prior to experimental treatments. For detection of native 5-bromo-2′-deoxyuridine (BrdU) foci, cells were seeded in media supplemented with 10 μg/ml BrdU 36 h prior to the experimental treatment. After the necessary treatments, they were incubated in the appropriate extraction buffer (Supplementary Table S6), rinsed twice in ice cold PBS, fixed in 2% paraformaldehyde in PBS for 20 min, and quenched for 10 min in 100 mM NH_4_Cl in PBS. Incubations in primary antibodies (Supplementary Table S4) were performed overnight at 4°C. Next, the cells were placed in PBS + 0.1% Tween-20 (PBST) for 5 min, rinsed six times in PBS and incubated with the appropriate secondary antibodies (Supplementary Table S5) diluted in PBS for 1 h at room temperature. The cells were then incubated in PBST containing 10 ng/μl DAPI for 20 min, rinsed six times in PBS and mounted on microscopy slides in 2% propyl gallate in PBS with 10% DMSO/80% glycerol as the solvent. Images were acquired on an upright fluorescence microscope (Zeiss AxioImager.Z1) with a Plan Apochromat 1.4 N.A. 63× oil immersion objective lens via MetaMorph (Molecular Devices, LLC) using a Prime 95B camera (Teledyne Photometrics). Scale bars in all micrographs represent 10 μm. Nuclear foci in fluorescence microscopy images were quantified by the Granularity application in MetaXpress 6 software (Molecular Devices, LLC) or using Imaris software (Oxford Instruments). All images within the same experiment were scaled evenly for brightness and contrast.

### Laser microirradiation

U-2 OS cells were cultured on 35-mm culture dishes containing a coverslip mounted on the bottom of the dish (MatTek Corporation) 48 h before the experiment and transfected with siRNA to CtIP. They were then transfected with the appropriate GFP-CtIP constructs ∼16 h before irradiation. Prior to imaging, the media was replaced with phenol-free DMEM supplemented with 2 mM l-glutamine, 10% FBS and 14 mM HEPES. Cells were then treated with 0.5 μg/ml Hoechst 33258 for 20–30 min, washed with PBS, then placed on the stage of a spinning disk (Ultraview, Perkin-Elmer) inverted microscope (Axiovert 200M, Carl Zeiss, with a 40×/1.3 N.A. Plan-Neofluar oil immersion objective lens) equipped with an electron-multiplying charge-coupled device (EM-CCD) camera (ORCA-FLASH-4.0; Hamamatsu Photonics). DSBs were generated along a 0.2–1 μm wide region across the nucleus of a single living cell by excitation of the Hoechst 33258 dye using a 405 nm laser line. The laser output was set to 10% (unless stated otherwise), and 10 iterations were used to generate localized DNA damage. GFP fluorescence imaging was recorded using 500–800 ms exposure times for 5–6 min using Volocity software (Perkin-Elmer). The mean accumulation ± standard deviation of GFP-CtIP from at least 24–48 cells pooled from three independent experiments was then plotted.

### Clonogenic survival assay

Parental or GFP-CtIP-expressing stable cell lines of U-2 OS were transfected with CtIP or non-targeting siRNA in two rounds, 24 h apart. ∼40 h after the first transfection, cells were seeded in duplicate into 6 cm dishes at ≥400 cells per dish and allowed to settle at 37°C for 6–8 h, after which they were treated with camptothecin at the indicated concentrations for 1 h at 37°C. The media in each dish was then replaced, and colonies were allowed to form over 9–11 days at 37°C. The colonies were fixed and visualized in 0.5% crystal violet/25% methanol. Colonies containing at least 50 cells were then scored and counted, and the surviving fraction was calculated accordingly (52).

### DNA fiber assay

U-2 OS cells were pulse-labeled with two thymidine analogs: first 20 μM 5-iodo-2′-deoxyuridine (IdU; Sigma-Aldrich), then 250 μM 5-chloro-2′-deoxyuridine (CldU; Sigma-Aldrich), each for 30 min at 37°C. Cells were washed twice with PBS after each pulse-labeling and then treated with 2 mM hydroxyurea (HU) for 4 h. The cells were then collected and resuspended in PBS at 100 000 cells/ml. 2 μl of the cell suspension was mixed with 10 μl of DNA Fiber Lysis Buffer (200 mM Tris–HCl pH 7.5, 50 mM EDTA, 0.5% SDS) on a glass slide. After  2 min, the slides were tilted at a 45° angle for spreading by gravity, and the resulting DNA spreads were air dried for 40 min, fixed in 3:1 methanol/acetic acid for 10 min and stored at 4°C. The DNA fibers were denatured with 2.5 M hydrochloric acid for 1 h, washed with PBS and blocked with 5% BSA in PBS containing 0.1% Tween-20 for 1 h. DNA immunostaining was then performed with a rat anti-BrdU/CldU antibody to detect CldU and a mouse anti-BrdU/IdU antibody to detect IdU (Supplementary Table S4) in a humidified chamber for 2 h at room temperature. The following secondary antibodies were then bound for 1 h at room temperature: chicken anti-rat—Alexa Fluor 488 and goat anti-mouse—Alexa Fluor 546 (Supplementary Table S5). The slides were air dried and mounted in ProLong Gold Antifade Mountant (Invitrogen, Thermo-Fisher). Images were sequentially acquired with an upright fluorescence microscope (Zeiss AxioImager.Z1) with a Plan Neofluar 1.3 N.A. 40× oil immersion objective lens via MetaMorph (Molecular Devices, LLC) using a Prime 95B camera (Teledyne Photometrics). The DNA tract lengths were measured with ImageJ software (version 1.51k), and the pixel length values converted into micrometers using the scale bars generated by the microscope. *n* ≥150 fiber tracts were scored for each data set. Scatterplots display the mean value and standard deviation.

### Protein expression and purification

The MRN (MRE11, RAD50 and NBS1) complex was expressed in *Sf*9 insect cells and purified according to an established protocol (53). DNA2 was expressed in *Sf*9 cells and purified as described (54). For two-step affinity purification of recombinant WT- and K578R-CtIP, *Sf*9 cells were infected with GST-CtIP-10XHis baculovirus. 72 h post-infection, cells were collected by centrifugation and the pellet was frozen on dry ice. To induce CtIP phosphorylation (pCtIP), the *Sf*9 culture was supplemented with 25 nM okadaic acid (Sigma) 4 h before harvest (i.e. 68 h after viral infection) followed by treatment with 1 μM camptothecin (Sigma) 1 h before harvest (i.e. 71 h after viral infection). Cells were lysed in Buffer 1 (1× PBS supplemented with 150 mM NaCl, 1 mM EDTA, 0.05% Triton X-100 and 1 mM DTT) supplemented with cOmplete protease inhibitor cocktail (Roche) and homogenized 10 times with a Dounce homogenizer (Pestle A). The cell lysate was incubated with 1 mM MgCl_2_ and 2.5 U/ml benzonase nuclease at 4°C for 1 h followed by centrifugation at 90 000g for 1 h. The soluble cell extract was then incubated with 1 ml of glutathione-sepharose beads for 90 min at 4°C with gentle rotation. The beads were washed twice with Buffer 1, and incubated with Buffer 2 (Buffer 1 with 5 mM ATP and 15 mM MgCl_2_) for 1 h at 4°C. The beads were then washed twice with Buffer 1 supplemented with 350 mM NaCl and once with P5 Buffer (50 mM sodium phosphate pH 7.0, 500 mM NaCl, 10% glycerol, 0.05% Triton X-100, 5 mM imidazole), then incubated with 60 U/ml PreScission protease (GE Healthcare Life Sciences) in P5 Buffer overnight at 4°C to cleave off the GST tag. Supernatant was then collected and completed to 10 ml with P5 Buffer before incubating for 1 h at 4°C with 400 μl of TALON bead slurry (Clontech) equilibrated in P5 Buffer. The TALON beads were washed twice with P30 buffer (50 mM sodium phosphate pH 7.0, 500 mM NaCl, 10% glycerol, 0.05% Triton X-100, 30 mM imidazole) before eluting the bound protein twice in one bead volume of P500 buffer (50 mM sodium phosphate pH 7.0, 500 mM NaCl, 10% glycerol, 0.05% Triton X-100, 500 mM imidazole). Eluted protein was then dialysed in the storage buffer (20 mM Tris pH 7.4, 200 mM NaCl, 10% glycerol, 1 mM DTT) and stored in aliquots at –80°C.

### MRN endonuclease assay

The dsDNA substrate used in this reaction was prepared as described (6). The MRN endonuclease assay was performed in a reaction buffer consisting of 25 mM MOPS pH 7.0, 60 mM KCl, 1 mM MnCl_2_, 5 mM MgCl_2_, 0.2% Tween-20, 2 mM DTT, 2 mM ATP and 100 nM of 5′-radiolabeled 70 bp dsDNA substrate blocked with 15 nM streptavidin for 5 min at room temperature. The indicated concentration of purified CtIP was added to the reaction and incubated at 37°C for 5 min. Where indicated, 20 nM of purified MRN was added and the reaction was allowed to proceed for a further 30 min at 37°C. Reactions were deproteinized in one-fifth volume of Stop Buffer (20 mM Tris–HCl pH 7.5, 2 mg/ml proteinase K) for 30 min at 37°C. An equal volume of 100% formamide was added to each reaction, and the samples were boiled at 95°C for 3 min before loading onto an 8% acrylamide/urea gel and running at 75 W for 60 min. The gel was dried on DE81 paper (Whatman) and signals were detected by autoradiography. Densitometric analyses were performed using the FLA-5100 phosphorimager (Fujifilm) and quantitated using the Image Reader FLA-5000 v1.0 software.

### DNA2 and CtIP nuclease assay

The nuclease assay was performed in a reaction buffer consisting of 25 mM MOPS pH 7.0, 60 mM KCl, 5 mM MgCl_2_, 0.2% Tween-20, 2 mM DTT, 2 mM ATP and 100 nM of 5′-radiolabeled flap DNA substrate. The indicated concentration of purified DNA2 or CtIP protein was added to the reaction and incubated at 37°C for 30 min followed by deproteinization in one-fifth volume of Stop Buffer (20 mM Tris–HCl pH 7.5, 2 mg/ml Proteinase K) for 30 min at 37°C. An equal volume of 100% formamide was added to each reaction, and the samples were boiled at 95°C for 3 min before loading onto an 8% acrylamide/urea gel and running at 75 W for 60 min. The gel was dried on DE81 paper (Whatman) and signals were detected by autoradiography. Densitometric analyses were performed using the FLA-5100 phosphorimager (Fujifilm) and quantitated using the Image Reader FLA-5000 v1.0 software. The flap DNA substrate was made by annealing JYM925 (GGGTGAACCTGCAGGTGGGCAAAGATGTCCTAGCAATGTAATCGTCAAGCTTTATGCCGT) and JYM926 (ACGCTGCCGAATTCTACCAGTGCCAGCGACGGACATCTTTGCCCACCTGCAGGTTCACCC).

### Image and data processing

Raw microscopic and immunoblot images were adjusted for brightness and contrast in Adobe Photoshop then arranged and labeled in Adobe Illustrator. Scale bars on micrographs represent 10 μm. Immunoblots were displayed avoiding saturation when possible. Graphs and scatterplots were generated in Prism (Graphpad) and display the mean and standard deviation (error bars). Two-tailed, unpaired, non-parametric Student's *t*-tests (Mann–Whitney) were performed in Prism to determine statistical significance. Asterisks depict statistically significant differences: ns (not significant), * (*P*$ \le$0.05), ** (*P*$ \le$0.01), *** (*P* < 0.001), **** (*P* < 0.0001). Schematic diagrams were prepared in Adobe Illustrator.

### Supplementary materials and methods

The Supplementary Information further details the siRNAs, DNA primers, antibodies, and extraction buffers for immunofluorescence staining used in this study.

## RESULTS

### SUMOylation events mediate homologous recombination

**Figure 1.**
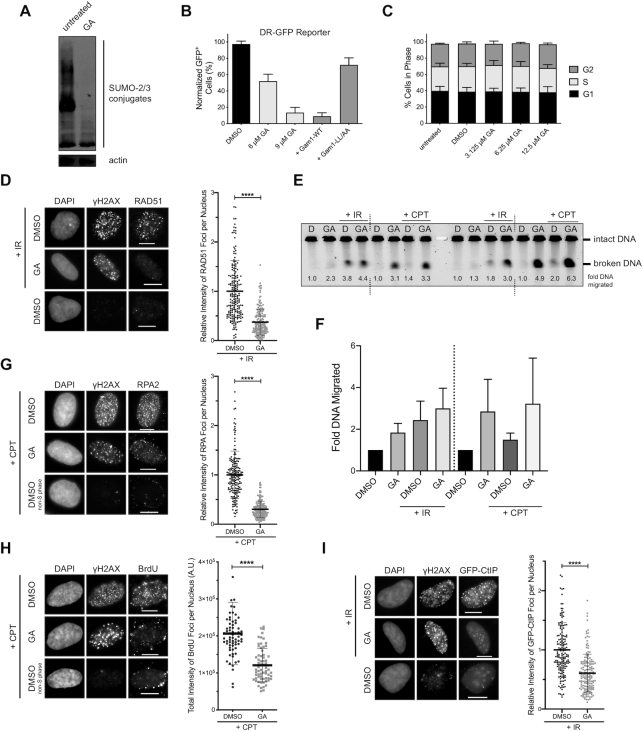
SUMOylation events mediate homologous recombination and DNA end resection. (**A**) U-2 OS cells were treated with ginkgolic acid 15:1 (GA) at 25 μM for 3 h. A reduction in SUMO-2/3 conjugates is seen upon GA treatment by immunoblot. (**B**) DR-GFP homologous recombination reporter assay in U-2 OS cells stably expressing the DR-GFP cassette. I-*Sce*I was expressed for 24 h in the presence of 0.025% DMSO (vehicle control), GA at the indicated concentrations, or co-expression of FLAG-tagged wildtype (WT) Gam1 or the LL/AA mutant. The means from two independent experiments are displayed. (**C**) U-2 OS cells were treated with 0.025% DMSO or GA at the indicated concentrations for 24 h and processed for DNA content analysis. Shown are the means from three independent experiments. (**D**) U-2 OS cells were pre-treated with 12 μM GA or 0.024% DMSO for 1 h, subjected to 10 Gy of IR or not, and recovered for 3 h in the presence of GA. Immunofluorescence (IF) micrographs presented are representative of at least four independent experiments (left panel). The total RAD51 foci intensity was quantified from ≥179 RAD51 foci-positive cells per condition from three independent experiments (right panel). (**E**) U-2 OS cells were pre-treated with 0.024% DMSO (D) or 12 μM GA (GA) for 2 h, then either exposed to 20 Gy of ionizing radiation (IR), treated with 1 μM CPT for 1 h in the presence of DMSO or GA, or not. 1 × 10^6^ cells per condition were embedded into agarose plugs and digested with Proteinase K. Each plug was cut in half, and both halves were resolved by pulsed-field gel electrophoresis. The representative gel shown has duplicate halves from one experiment run simultaneously (left and right). Intensities of the migrated and immobile DNA bands were quantified and used to calculate relative DNA migration, which is presented below each lane and indicates the quantity of DSBs induced. (**F**) Quantification of relative DNA migration from the gel in (E), averaged with data from a second independent replicate. (**G** and **H**) Left panels: IF micrographs of U-2 OS cells pre-treated with 12 μM GA or 0.024% DMSO for 2 h, after which 1 μM camptothecin (CPT) was added for an additional hour. As only cells in S phase are sensitive to CPT, cells that did not respond to CPT are also presented. Right panels: In (G), ≥178 γH2AX^+^ cells per condition from three independent experiments were quantified for total RPA2 foci intensity. In (H), ≥66 γH2AX^+^ cells per condition from one experiment were quantified for total BrdU foci intensity. Cells in (H) were cultured in BrdU-containing media prior to treatment. (**I**) IF micrographs (left panel) of U-2 OS cells stably expressing GFP-CtIP pre-treated with 12 μM GA or 0.024% DMSO for 1 h, subjected to 10 Gy of IR or not, and recovered for 4 h in the presence of GA. Right panel: the total GFP-CtIP foci intensity was quantified from ≥158 GFP-CtIP foci-positive cells per condition from three independent experiments. Micrographs in (G–I) are representative of the results from at least six independent experiments.

Currently, the role of SUMOylation in the regulation of homologous recombination (HR) proteins is not well understood. We thus sought to identify potential SUMO targets in the HR pathway and characterize how SUMOylation could affect their function. To screen for SUMO targets, we utilized ginkgolic acid 15:1 (GA), an inhibitor of global protein SUMOylation that interferes with formation of the E1-SUMO intermediate and does not affect ubiquitylation (55). We chose to block SUMOylation with acute GA treatment as opposed to short interfering RNA (siRNA)-mediated depletion of UBC9 (the sole E2 enzyme in the SUMOylation cascade) to avoid issues associated with RNA interference, namely incomplete knock-down, off-target effects and cellular adaptation mechanisms from prolonged knockdown. We first confirmed that treating U-2 OS cells with GA was able to reduce the presence of higher-order SUMO conjugates via immunoblot (Figure 1A), validating the effectiveness of the inhibitor. To determine if SUMOylation events mediate the process of HR, we performed a gene conversion assay in U-2 OS cells stably expressing the DR-GFP reporter construct (U-2 OS DR-GFP) (56) in the presence or absence of GA. Expressing I-*Sce*I endonuclease in these cells generates a site-specific DSB within the reporter construct, which when repaired by HR results in expression of a functional green fluorescent protein (GFP) product (Supplementary Figure S1A). The number of GFP^+^ cells, measured by flow cytometry, was used as a readout of the frequency of HR events. Indeed, treating the cells with GA reduced the number of GFP^+^ cells in a dose-dependent manner relative to cells treated with vehicle control (DMSO) (Figure 1B). Since HR is active during the S and G_2_ stages of the cell cycle, we wondered if treating cells with GA could be altering the cell cycle distribution. To address this, we treated U-2 OS cells with GA and quantified their DNA content by propidium iodide staining and flow cytometry. The chosen concentrations of GA did not drastically alter the proportion of cells within G_1_, S and G_2_ phase (Figure 1C), confirming that the inhibition of HR in U-2 OS DR-GFP was not due to GA biasing cells to the G_1_ stage. To verify that the GA-dependent reduction in HR frequency was due to the inhibition of SUMOylation, we used another inhibitor of SUMOylation, the avian adenoviral protein Gam1 (57,58). Mechanistically, Gam1 inhibits SUMOylation by binding to the SUMO E1 heterodimer SAE1/SAE2 and recruiting it to an elongin/cullin E3 ubiquitin ligase complex, targeting it for proteasomal degradation. Gam1 expression also promotes proteasomal degradation of the SUMO E2 UBC9 (59,60), and all these activities are abrogated in the L258A/L265A (LL/AA) mutant of Gam1 (58–60). After confirming that Gam1 expression was able to reduce protein levels of UBC9 (Supplementary Figure S1B), and only slightly increased the proportion of cells in G_1_ phase (Supplementary Figure S1C), we co-expressed in U-2 OS DR-GFP cells I-*Sce*I with either wildtype (WT)- or LL/AA-Gam1. Expressing WT-, but not LL/AA-Gam1, led to a reduction in the frequency of HR similar to treatment with GA (Figure 1B). Hence, HR is regulated by SUMOylation events, as it can be inhibited both by GA treatment and by Gam1 expression.

### DNA end resection and CtIP recruitment are regulated by SUMOylation events

We next sought to map out which players in the HR pathway were potentially impacted upon inhibition of SUMOylation by GA. We reasoned that certain disruptions in function could be visualized by defects in the accumulation of HR proteins at sites of DNA damage in response to camptothecin (CPT) or ionizing radiation (IR). Starting at the assembly of RAD51 nucleofilaments, a downstream event in HR, we observed that GA treatment inhibited the formation of RAD51 foci in U-2 OS cells in response to IR, despite the cells still incurring DSBs, as visualized by the formation of γH2AX foci (Figure 1D). This validated that HR as a process was inhibited by GA, agreeing with the reduction in HR frequency seen in the U-2 OS DR-GFP reporter cells (Figure 1B). As H2AX is a substrate for SUMOylation itself (61), γH2AX foci intensity could be altered in the presence of GA and not reflect the true extent of the DNA damage load. We thus resorted to using pulsed-field gel electrophoresis (PFGE) to measure the degree of DSBs induced by IR in the presence or absence of GA. Interestingly, GA alone and in combination with IR exacerbated the proportion of broken DNA in U-2 OS cells (Figure 1E and F), suggesting more DSBs were in fact induced with GA present. Thus the reduction in RAD51 foci observed with GA treatment reflects a true inhibition in RAD51 function, as the GA-treated cells had incurred even more DSBs than those treated with vehicle control. We then hypothesized that the reduction in RAD51 foci could, at least in part, be a result of hindered DNA end resection, an event upstream of RAD51 filament assembly. End resection generates single-stranded DNA (ssDNA) overhangs that are protected from nucleolytic degradation by the recruitment of RPA complexes (4), so we chose to detect both RPA and ssDNA foci as readouts of functional end resection. Treating U-2 OS cells with GA reduced the intensity of RPA foci formed in response to CPT (Figure 1G), and reduced CPT-induced 5-bromo-2′-deoxyuridine (BrdU) foci detected under non-denaturing conditions (Figure 1H, Supplementary Figure S1D), exposure of which represents ssDNA. As a control, by PFGE, we detected more DSB damage inflicted in cells treated with CPT and GA than CPT alone (Figure 1E and F), emphasizing the true reduction in RPA and BrdU foci seen upon GA treatment. In line with this, expression of Gam1 also strongly inhibited the formation of RPA (Supplementary Figure S1E) and native BrdU foci (Supplementary Figure S1F). Although transient Gam1 expression slightly increased the proportion of cells in G_1_ (Supplementary Figure S1C), our use of CPT as a damaging agent ensured damage was only inflicted on cells within S phase (62). Together, our findings suggest end resection is dependent on SUMOylation events, which, when inhibited, result in less ssDNA and reduced RPA recruitment to sites of damage. We hypothesized that this impairment in end resection could be due to defects in the recruitment of the end resection machinery to DSBs. We proceeded by examining the ability for the MRN complex components NBS1 and MRE11 as well as MRN co-factor CtIP (6) to form IR-induced foci in the presence of GA, selecting U-2 OS cell lines stably expressing GFP-tagged versions of CtIP and MRE11 to improve detection of IR-dependent foci over background. While the intensity and number of MRE11-GFP (Supplementary Figure S1G) and NBS1 (Supplementary Figure S1H) foci were not substantially altered by GA treatment, the intensity of GFP-CtIP foci was notably inhibited in the presence of GA (Figure 1I), suggesting less CtIP was being recruited to DSB sites upon inhibition of SUMOylation. Thus, we conclude end resection is dependent on SUMOylation, and one explanation may lie in a defect in CtIP recruitment to DSB sites upon shutdown of SUMOylation.

Since CtIP has also been found to function at the replication fork (24–28), we tested if conditions that induce replication stress also promoted the formation of CtIP foci. Treating U-2 OS cells with CPT at low concentrations (63) and the deoxynucleotide triphosphate (dNTP) pool-depleting agent hydroxyurea (HU) (64), both of which induce replication stress, led to the recruitment of CtIP into distinct foci in a dose-dependent manner (Supplementary Figure S2A). Interestingly, treatment with GA reduced the intensity of these foci, suggesting CtIP recruitment to sites of replication stress is also mediated by SUMOylation processes (Supplementary Figure S2B). In summary, CtIP forms foci both when DSBs and replication stress are induced, and in both cases this recruitment is dependent on SUMOylation events.

### CtIP is a target for SUMO-2 modification

**Figure 2.**
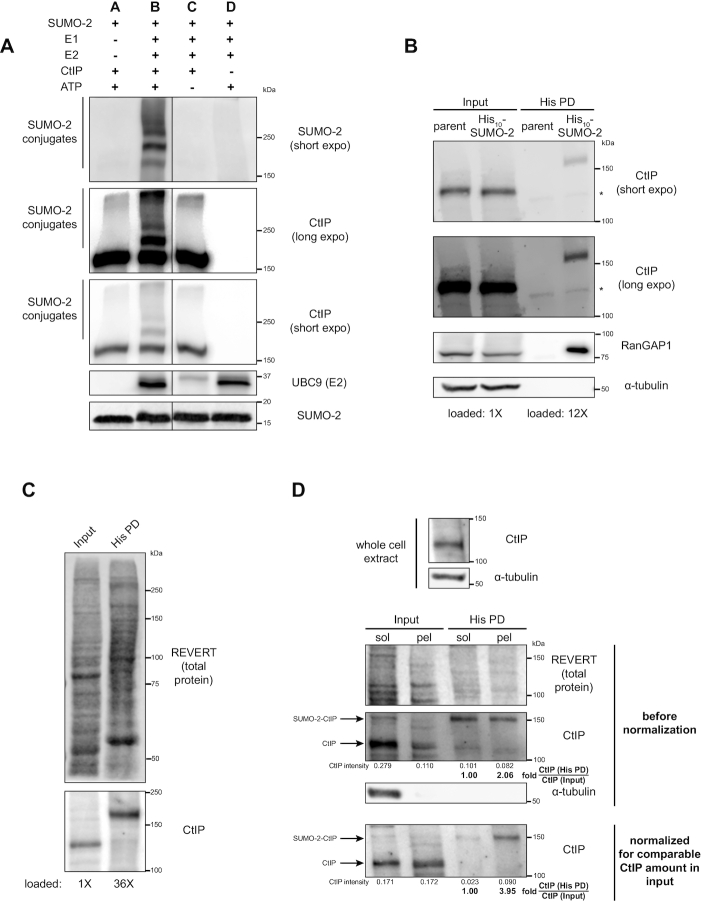
CtIP is a target for SUMO-2 modification. (**A**) *In vitro* reactions assembled using a SUMO-2 conjugation kit and recombinant glutathione S-transferase (GST)-tagged human CtIP were resolved by SDS-PAGE and immunoblotted. Despite even input of UBC9 into the reactions, less UBC9 was consistently detected by immunoblot in Reaction C; we speculate the antibody used preferentially recognizes auto-SUMOylated UBC9, which is absent when ATP is eliminated from the reaction (Reaction C). The solid line defines where an intervening lane was spliced out of the image. The immunoblot shown is representative of six independent experiments. (**B**) HeLa cells expressing 10XHis-tagged SUMO-2 (His_10_-SUMO-2) or parental HeLa cells were portioned into input and His pull-down (His PD) fractions and processed as whole cell extracts or Ni-NTA affinity purifications, respectively, then resolved by SDS-PAGE. Shown is a representative result of two independent experiments. * indicates a non-specific immunoreactive band. Corresponds to Supplementary Figure S3B. (**C**) As in (B) but with His_10_-SUMO-2 cells only, representative of at least three independent experiments. (**D**) 10% of a cell pellet of HeLa His_10_-SUMO-2 cells was lysed as the whole cell extract (top panel); the remainder was fractionated for chromatin enrichment. 1/9 of each of the resulting soluble (sol) and chromatin-enriched pellet (pel) fractions was saved to represent the input prior to His PD. The remainder was processed for His PD, and all fractions were resolved by SDS-PAGE. For the first run (middle panel), the His PD fractions contain 32X more of the starting amount of sample relative to the input. In the second run (bottom panel), the pellet fraction (for input and His PD portions) was loaded to represent a similar starting amount of CtIP as the soluble fraction. Shown is a representative result of two independent experiments.

With this evidence as groundwork, we hypothesized that CtIP is a substrate for SUMOylation. To examine if CtIP is a potential substrate for SUMOylation, we performed an *in vitro* SUMOylation assay (Figure 2A). Indeed, recombinant CtIP was modified by multiple SUMO-2 moieties in the presence of SUMO E1 SAE1/SAE2 and E2 UBC9, and the reaction was dependent on ATP. We then moved to detect and characterize CtIP SUMO modification *in vivo*. To enable this, we obtained HeLa cells stably expressing decahistidine-tagged SUMO-2 (HeLa His_10-_SUMO-2) at modest levels (50,65). The presence of the His_10_-tag and slight increase in expression of SUMO-2 in HeLa His_10-_SUMO-2 cells was confirmed via immunoblot relative to the parental HeLa cells (Supplementary Figure S3A). Conceptually, the expression of His_10_-tagged SUMO-2 allows all SUMO-2 conjugates in the cell to be labeled with the His_10_ tag. Lysis of the cells under strongly denaturing conditions (6 M guanidium-HCl) prevents reversal of SUMOylation by denaturing SUMO proteases, while subsequent nickel affinity purification isolates the fraction of cellular proteins that are His_10_-tagged SUMO-2 conjugates (51). We first verified that Ni-NTA purification (‘His pull-down’, or His PD) of HeLa His_10-_SUMO-2 lysates was able to enrich for His_10_-SUMO-2 conjugates (Supplementary Figure S3B). Next, to detect if CtIP is SUMOylated *in vivo*, we split asynchronous HeLa and HeLa His_10-_SUMO-2 cells into two fractions, one which was processed as whole cell extract, while the remainder was processed for Ni-NTA purification. SDS-PAGE and immunoblotting of both fractions revealed that CtIP was detected in the His pull-down fraction in HeLa His_10-_SUMO-2 cells but not parental HeLa cells, despite similar levels of expression in the whole cell extract (Figure 2B), as was the case for RanGAP-1, a known target for SUMOylation (51). Moreover, CtIP in the His pull-down fraction exhibited a slower electrophoretic migration (near 150 kDa) compared to CtIP in the whole cell extract (∼125–130 kDa) (Figure 2C), the increase in molecular weight supportive of the linkage of one or two SUMO-2 moieties onto the protein (51). Subsequent densitometric quantification of CtIP in the His pull-down fraction versus whole cell extract suggested only 2.5–5% of endogenous CtIP was modified by SUMO-2 at the steady state, reflecting the low abundance of the modified form. Supportive that the ∼150 kDa species observed was indeed SUMO-2-modified CtIP, we first found that the signal intensity could be reduced in a dose-dependent manner in the presence of GA (Supplementary Figure S3C). Second, to ensure the immunoreactive bands detected were in fact CtIP, we validated the specificity of our CtIP antibody. The antibody could not detect a truncated CtIP mutant missing residues 732–892 (D6) (47) (Supplementary Figure S3D), consistent with it being raised to bind the CtIP C-terminus (66). Thus, CtIP is a target for SUMOylation in the cell, with a small fraction of CtIP being modified by SUMO-2 *in vivo*.

We next characterized if SUMOylated CtIP was localized to chromatin. HeLa His_10-_SUMO-2 lysates were separated into chromatin-enriched and soluble fractions which were then subjected to His pull-down to enrich for SUMO-2 modified proteins. We observed that the majority of CtIP partitioned into the soluble fraction. Although a small fraction of CtIP remained chromatin-bound, this fraction was enriched for SUMOylated species (Figure 2D). This suggests that SUMOylated CtIP is bound to chromatin, indicating that SUMOylation of CtIP may be important for its function, either allowing it to be targeted to chromatin, or being SUMOylated once it is recruited to chromatin. In summary, CtIP is a SUMO-2 substrate both *in vitro* and *in vivo*, and SUMO-2-modified CtIP is enriched on chromatin.

### Analysis of CtIP SUMOylation status in S phase and in response to double strand breaks and replication stress

**Figure 3.**
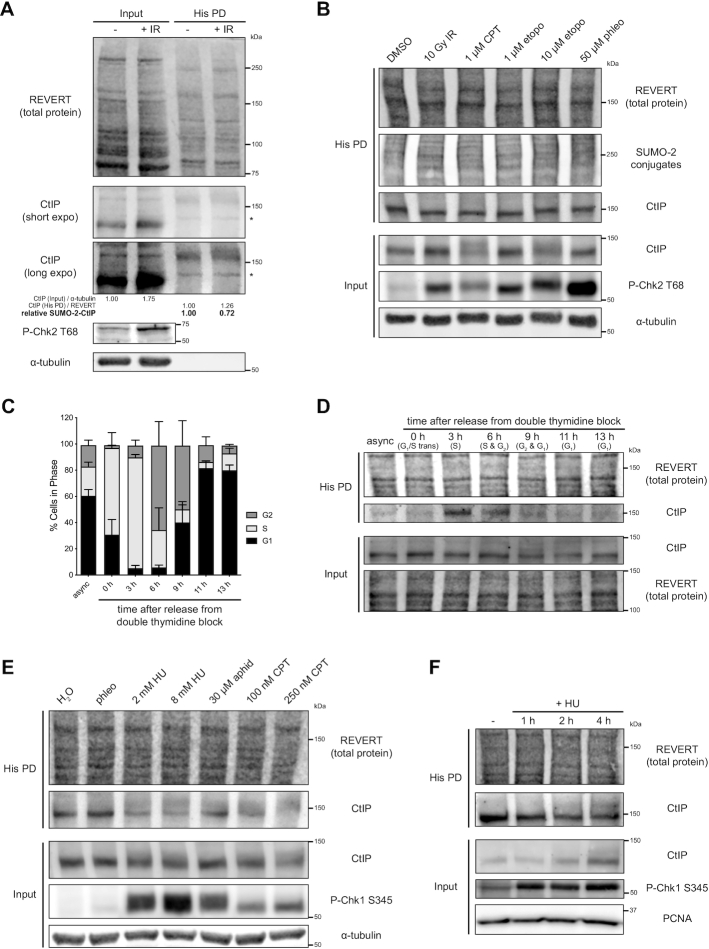
Analysis of CtIP SUMOylation status in S phase and in response to double-strand breaks and replication stress. (A, B, D, E, F) HeLa His_10_-SUMO-2 cells were treated as indicated, portioned into input and His PD fractions and processed accordingly before SDS-PAGE and immunoblotting. Chk2 and Chk1 phosphorylation were used as readouts for the induction of DSBs or replication stress, respectively. (**A**) Asynchronous cells were subjected to 10 Gy of IR or not and allowed to recover for 1 h. Shown is a representative result of three independent experiments. * indicates a non-specific immunoreactive band. Corresponds to Supplementary Figure S4A. (**B**) Asynchronous cells were exposed to 10 Gy of IR and allowed to recover for 1 h, or subject to CPT, etoposide (etopo), or phleomycin (phleo) at the indicated concentrations for 1 h. Shown is a representative result of four independent experiments. CtIP typically exhibits smearing upon treatment with high dose CPT (67) and etoposide (input fraction). (**C**) HeLa His_10_-SUMO-2 cells were left asynchronous (async) or synchronized by double thymidine block and released for various timepoints to reach different cell cycle phases, then a portion was processed for DNA content analysis. Shown are the means from three independent experiments. (**D**) As in (C), but this time processed for immunoblot of input and His PD fractions. Shown is a representative result of three independent experiments. (**E**) Asynchronous cells were subject to 25 μM phleomycin for 1 h or the indicated replication stress inducing agents for 4 h. 0.8% H_2_O served as the vehicle control; aphidicolin (aphid); hydroxyurea (HU). Shown is a representative result of three independent experiments. (**F**) Asynchronous cells were treated with 2 mM HU for the indicated timepoints or not treated for 4 h. Shown is a representative result of at least three independent experiments.

Having detected low abundance CtIP SUMOylation, we proceeded to investigate if the modification could be enhanced in response to DNA damage. As CtIP plays a crucial role in the repair of DSBs (5), we tested if its SUMOylation would increase in response to DSBs, similar to BRCA1, which is SUMOylated in response to IR (67). Exposure to IR did not drastically increase the degree of SUMOylated CtIP as detected by His pull-down (Figure 3A), despite equal pull-down efficiency between the Ni-NTA purification samples, as detected by the reversible total protein stain REVERT. Nor was SUMOylation status noticeably altered in response to IR for the end resection factors MRE11 and NBS1, although there was a noticeable induction for BRCA1 SUMOylation as expected (67) (Supplementary Figure S4A). Similarly, treatment with other DSB inducing agents including CPT, etoposide and phleomycin did not increase levels of SUMO-2 CtIP (Figure 3B). We reasoned that the lack of response in CtIP was because our experiments had been conducted on asynchronous cells, which are primarily in G_1_ phase (Figure 3C), and that if CtIP SUMOylation was involved in end resection as a part of HR that it would be more apparent in cells in the S and G_2_ phases, when HR occurs. As such, we synchronized HeLa His_10-_SUMO-2 cells to the G_1_/S transition via double thymidine block. The cells were then collected after being released from thymidine block for various times, allowing them to progress to different stages of the cell cycle. Samples collected after each timepoint were fractioned and processed either for His pull-down or as whole cell extracts, or for DNA content analysis by propidium iodide staining. DNA content analysis confirmed the cells had indeed been synchronized to various cell cycle stages (Figure 3C), and immunoblot analysis demonstrated no substantial changes in His_10-_SUMO-2 expression levels over the phases (Supplementary Figure S4B). Intriguingly, the degree of CtIP modified by SUMO-2 was dependent on the cell cycle stage, increasing and peaking considerably at S phase (3 h post-release) while decreasing as the cells progressed through G_2_ and reaching a minimum in G_1_ phase, despite less drastic fluctuations in total CtIP expression over the cell cycle (Figure 3D and Supplementary Figure S4C). This induction of CtIP SUMOylation reflected a ∼2–3-fold increase in SUMO-2-CtIP compared to asynchronous cells. Observing the large impact the cell cycle stage can have on SUMOylation, we next tested if cells synchronized to S or G_1_ phase would respond to DSBs by further upregulating CtIP SUMOylation. Still, inducing DSBs via IR did not increase CtIP SUMOylation, whether the cells were synchronized to S or G_1_ phase (Supplementary Figure S4D). The induction of SUMOylation in S phase suggested that CtIP was SUMOylated in response to active DNA replication or replication stress. We consequently examined if there were alterations in CtIP SUMOylation in response to externally applied replication stress. His pull-down experiments on asynchronous cells revealed a surprising reduction in SUMO-2-CtIP upon treatment with HU, aphidicolin (a DNA polymerase inhibitor), and low concentration CPT, all inducers of replication stress, despite steady expression levels of CtIP (Figure 3E). In support of this, we observed a time-dependent reduction in SUMO-2-CtIP over 4 h of HU treatment (Figure 3F). Thus, CtIP modification by SUMO-2 occurs constitutively in S phase, and this modification is not detectably induced by DSB damage, but is reduced during exogenous replication stress.

### SUMOylation of CtIP in S phase is dependent on cyclin-dependent kinase and ATR activities and an interaction with PCNA

**Figure 4.**
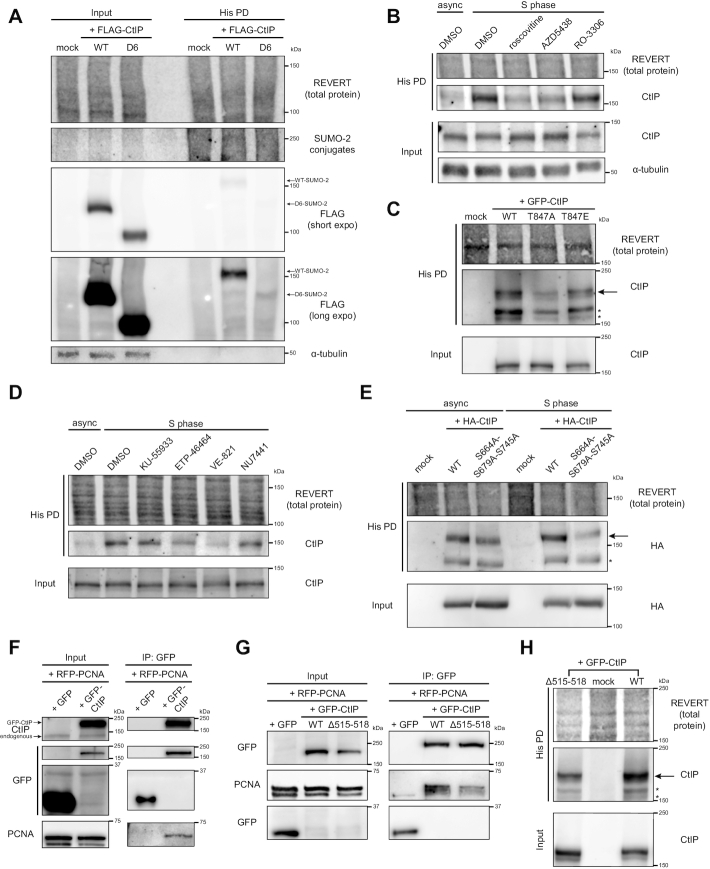
SUMOylation of CtIP in S phase is dependent on cyclin-dependent kinase and ATR activities and an interaction with PCNA. (A–E, H) HeLa His_10_-SUMO-2 cells were treated as indicated, portioned into input and His PD fractions and processed accordingly before SDS-PAGE and immunoblotting. * indicates an exogenous CtIP immunoreactive band that is not of interest; we speculate it is either lower molecular weight SUMO-2-modified CtIP or unmodified tagged-CtIP retained in the His PD fraction due to overexpression. (**A**) HeLa His_10_-SUMO-2 cells were transfected with FLAG-tagged wildtype (WT) CtIP or a C-terminal truncation mutant (D6) (see Supplementary Figure S7A) or mock transfected. Shown is a representative result of four independent experiments. (**B**) HeLa His_10_-SUMO-2 cells were synchronized by double thymidine block and released to mid-S phase for 1 h in plain media, then for 2.5 h in the presence of 0.1% DMSO (vehicle control), 25 μM roscovitine, 2.5 μM AZD5438, or 10 μM RO-3306. Asynchronous cells (async) were treated in 0.1% DMSO for 2.5 h. Shown is a representative result of three independent experiments. (**C**) HeLa His_10_-SUMO-2 cells were synchronized to mid-S phase. 24 h prior to harvest, they were transfected with GFP-CtIP-WT or substitution mutants at residue T847, or mock transfected. Shown is a representative result of two independent experiments. (**D**) HeLa His_10_-SUMO-2 cells were left asynchronous or synchronized by double thymidine block and released for 3 h to mid-S phase in the presence of 0.2% DMSO, 10 μM KU-55933 (ATM inhibitor), 20 μM ETP-46464 or 20 μM VE-821 (ATR inhibitors), or 1 μM NU-7441 (DNA-PKcs inhibitor). Shown is a representative result of at least four independent experiments. (**E**) HeLa His_10_-SUMO-2 cells were cells were left asynchronous or synchronized to mid-S phase. 24 h prior to harvest, they were transfected with HA-tagged WT-CtIP or an alanine substitution mutant at residues S664, S679 and S745, or mock transfected. Shown is a representative result of two independent experiments. (**F**) U-2 OS cells were co-transfected with RFP-PCNA and either GFP empty vector or GFP-CtIP and processed for immunoprecipitation (IP) of GFP. Prior to IP, a portion of lysate was saved as an input control. (**G**) As in (F), except cells were depleted of endogenous CtIP by siRNA, then co-transfected with RFP-PCNA and either GFP empty vector, GFP-CtIP-WT or -Δ515–518. Shown is a representative result of at least six (F) or three (G) independent experiments. (**H**) HeLa His_10_-SUMO-2 cells were synchronized to mid-S phase. 24 h prior to harvest, they were transfected with GFP-CtIP-WT or the Δ515–518 deletion mutant. Shown is a representative result of two independent experiments.

As we only observed an induction of CtIP SUMOylation during S phase, we focused on characterizing this occurrence, seeking to identify factors that mediate it. We first examined the C-terminus of human CtIP, which contains a Sae2-like domain evolutionarily conserved among CtIP orthologues in vertebrates and in budding and fission yeast (5) (Supplementary Figure S5A). To determine if the residues in the C-terminus could potentially play a role in the ability for CtIP to be SUMOylated, we compared the ability of full length (WT) and C-terminally-truncated CtIP (D6, missing residues 732–892) to be modified by SUMO-2. FLAG-tagged-WT- and D6-CtIP were transiently expressed in S phase synchronized HeLa His_10-_SUMO-2 cells and processed for His pull-down. While both constructs were expressed at similar levels, SUMOylation of the D6 mutant was almost abolished relative to WT-CtIP (Figure 4A). While we could not rule out that certain lysine residues within the region deleted in D6 (161 residues) were SUMOylation sites or if the D6 mutant was altered in its ability to interact with DNA (68,69), one explanation could be that residues within the C-terminus, upon modification by phosphorylation, could be promoting CtIP’s SUMOylation. Indeed, the C-terminus of CtIP contains serine and threonine residues that are sites of phosphorylation and regulate the function of CtIP in HR repair (35–37) (Supplementary Figure S5A). Kinases that target these sites include the DNA damage sensing kinases ATM and ATR, which control the cellular response to DNA damage and replication stress, respectively (70), and the cyclin-dependent kinases (CDKs), which govern temporal progression of the cell cycle (71). Given that CtIP SUMOylation occurs during S phase, and that the C-terminus contains several CDK sites (35,37), we predicted that inhibiting the activity of CDKs would block bulk CtIP SUMOylation. In agreement, HeLa His_10-_SUMO-2 cells synchronized to S phase and treated with two broad-acting CDK inhibitors that inhibit CDK2, which mediates progression through late G_1_ and S phase (72), roscovitine and AZD5438 (73,74), exhibited reduced CtIP SUMOylation compared to the vehicle control (Figure 4B). We verified the acute treatments had little immediate impact on the cell cycle distribution and that the inhibitors were active (Supplementary Figure S5B, C). Interestingly, RO-3306, an inhibitor specific to CDK1, a CDK active in the transition from late G_2_ to mitosis (75), exhibited much less of an inhibitory effect (Figure 4B). This suggests CDKs other than CDK1, perhaps CDK2, may be phosphorylating CtIP and facilitating its SUMOylation. We further confirmed the role of CDK activity in CtIP SUMOylation by obtaining mutants of GFP-tagged CtIP at a conserved CDK site, T847 (Supplementary Figure S5A), whose phosphorylation promotes DNA end resection (35,36). We first verified that GFP-WT-CtIP could indeed be SUMOylated itself *in vivo*, as a clear shift in molecular weight was observed when the construct was expressed in HeLa His_10-_SUMO-2 cells and subjected to His-tag purification (Supplementary Figure S5D). The detected immunoblot signal was specific to the CtIP portion of the construct, since it was not seen when GFP empty vector was transfected instead. To test the role of T847 in CtIP SUMOylation, GFP-CtIP-WT or mutants where T847 was substituted with alanine (loss of function, T847A) or glutamate (phosphomimic, T847E) were expressed in HeLa His_10-_SUMO-2 cells synchronized to S phase. By His-tag pull-down, the T847A mutant had partially reduced SUMOylation compared to WT-CtIP, and this was partially restored in the T847E mutant (Figure 4C). Thus, phosphorylation of CtIP by CDKs, at T847 and potentially other CDK sites, mediates its SUMOylation in S phase.

As the C-terminus of CtIP also contains residues that are targets for phosphorylation by ATM and ATR (33–37), we next pursued if the activities of these kinases could be prerequisites for CtIP SUMOylation. To address this, HeLa His_10-_SUMO-2 cells were synchronized to S phase while being treated acutely with inhibitors to ATM (KU-55933), ATR (ETP-46464 and VE-821) and the related DNA-dependent protein kinase catalytic subunit (DNA-PKcs) (NU-7441), and SUMOylated CtIP was isolated by His pull-down. Importantly, we verified that the chosen compounds only exerted minimal effects on the cell cycle profile (Supplementary Figure S5E), and were effective at inhibiting their target kinases (Supplementary Figure S5F–I). ETP-46464 and VE-821 treatment reduced CtIP SUMOylation (Figure 4D), suggesting that ATR-dependent phosphorylation events on CtIP could be regulating its SUMOylation. To confirm this, we obtained an HA-tagged CtIP mutant where three previously identified S/TQ sites (the consensus sequence phosphorylated by ATM and ATR (76)), S664, S679 and S745 (33,34,37) (Supplementary Figure S5A), were mutated to alanine (48). In HeLa His_10-_SUMO-2 cells, exogenous HA-CtIP-WT was SUMOylated more than its S664A/S679A/S745A counterpart (Figure 4E). This was seen in both asynchronous and S phase-synchronized cells, with the difference more obvious in cells in S phase, supporting the cell cycle-dependent nature of CtIP SUMOylation. Another S/TQ site on CtIP is T859 (Supplementary Figure S5A), whose phosphorylation by ATR upon DSB formation allows CtIP to bind to chromatin and activate end resection (36). Bulk SUMOylation of CtIP in S phase was not impacted in a mutant with T859 mutated to alanine, T859A (Supplementary Figure S5J), indicating this particular site is dispensable for the CtIP SUMOylation observed. Thus, ATR-dependent phosphorylation of CtIP, potentially at residues S664A, S679A and/or S745A, but not at T859, mediates constitutive CtIP SUMOylation in S phase.

Our observations that CtIP forms foci during replication stress, and is SUMOylated during S phase in a CDK- and ATR-dependent manner, are compatible with the finding that CtIP interacts with the DNA polymerase processivity factor PCNA (25). The study found a putative PCNA-interacting protein domain (PIP-Box (77)) within CtIP at residues 518–537 (Supplementary Figure S5A). This PIP-Box resides in a region of CtIP dubbed the Replication Foci Targeting Sequence (RFTS, residues 505–546) (25). The RFTS was found to be sufficient for binding PCNA as well as targeting CtIP to BrdU^+^ foci of active DNA replication. Deleting residues 515–518 near and within the PIP-Box disrupted the interaction of the RFTS fragment with PCNA, and prevented RFTS from being targeted to replication foci (25). We hypothesized that the interaction between PCNA and CtIP facilitates its SUMOylation, perhaps by helping recruit CtIP to sites of DNA replication or replication stress. We first verified that both proteins do interact as reported. We co-expressed monomeric red fluorescent protein (RFP)-tagged PCNA and GFP-CtIP in U-2 OS cells and performed co-immunoprecipitation (Co-IP) experiments. Immunoprecipitating GFP-CtIP co-purified RFP-PCNA (Figure 4F), and *vice versa* (Supplementary Figure S6A), confirming the two proteins associate with each other. By Co-IP, we also found that the Δ515–518 mutation in CtIP (Supplementary Figure S5A) could partially disrupt its interaction with PCNA (Figure 4G), in support of the previous findings (25). To study the impact of disrupting the PCNA-CtIP interaction on CtIP SUMOylation, we expressed in S phase-synchronized HeLa His_10-_SUMO-2 cells GFP-CtIP-Δ515–518. The His pull-down assay revealed the Δ515–518 mutant was markedly less SUMOylated than GFP-CtIP-WT (Figure 4H), suggesting the interaction with PCNA promotes CtIP SUMOylation. In summary, we have shown that conjugation of SUMO-2 to CtIP in S phase is promoted by residues in its C-terminus, particularly those that are targets of the activities of the CDKs and ATR kinase, as well as residues 515–518, which may act by enhancing an interaction with PCNA.

### SUMOylation of CtIP in S phase is dependent on the E3 SUMO ligase PIAS4

**Figure 5.**
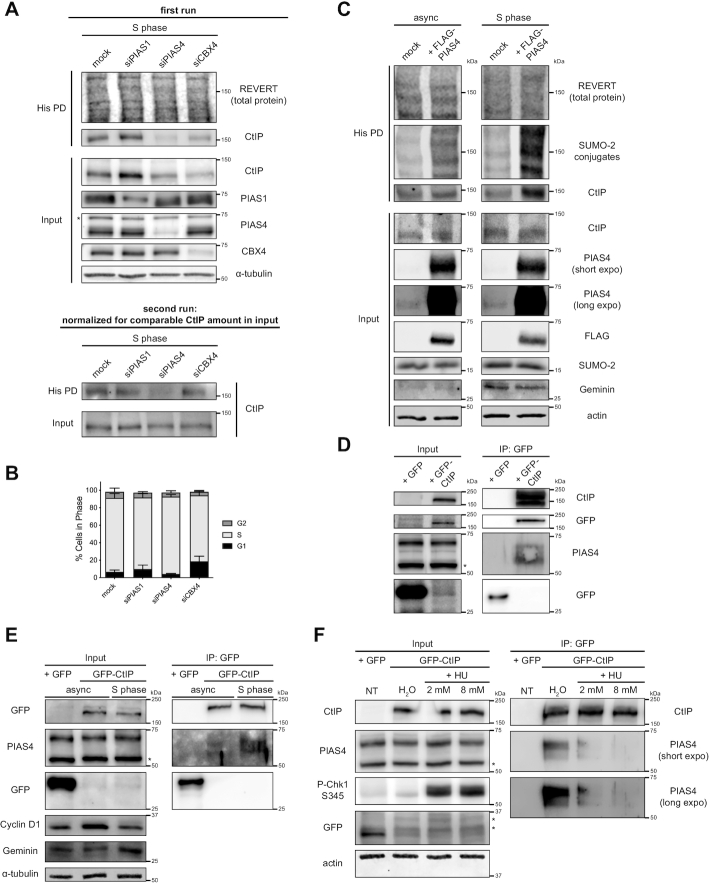
SUMOylation of CtIP in S phase is dependent on the E3 SUMO ligase PIAS4. (**A**) HeLa His_10_-SUMO-2 cells were transfected twice 24 h apart with siRNAs targeting either PIAS1 (at 50 nM), PIAS4 (at 40 nM), or CBX4 (at 40 nM), or mock transfected, while being synchronized by double thymidine block. 48 h after the first transfection, the cells were released for 3 h to approach mid-S phase, portioned into input control and His PD fractions, and processed for whole cell lysis or Ni-NTA affinity purification (top panel). A second run (bottom panel) was performed where the input and His PD portions were normalized to accommodate for alterations in CtIP expression resulting from the depletion of the various E3 SUMO ligases. Shown is a representative result of three independent experiments. (**B**) As in (A), but using the portion of cells processed for DNA content analysis. Shown are the means of three independent experiments. (**C**) HeLa His_10_-SUMO-2 cells were left asynchronous or synchronized to S phase. 24 h prior to harvest, they were transfected with FLAG-tagged human PIAS4 or mock transfected. The cells were portioned and processed as input controls or for His PD. Shown is a representative result of two independent experiments. (**D**) U-2 OS cells were transfected with GFP-CtIP or GFP empty vector and processed for IP of GFP. Prior to IP, a portion of lysate was saved as an input control. Shown is a representative result of at least four independent experiments. (**E**) U-2 OS cells stably expressing GFP-CtIP were left asynchronous or synchronized to S phase and processed for IP of GFP. U-2 OS cells transfected with GFP empty vector served as a control. Shown is a representative result of at least four independent experiments. (**F**) Asynchronous U-2 OS cells stably expressing GFP-CtIP were treated for 4 h with HU at the indicated concentrations or 0.8% H_2_O (vehicle control) and processed for IP of GFP. U-2 OS cells transfected with GFP empty vector served as a control. Shown is a representative result of at least four independent experiments. * indicates a non-specific immunoreactive band. Geminin and Cyclin D1 are markers for S and G1 phases, respectively.

Next, we investigated which E3 SUMO ligase(s) could be SUMOylating CtIP in S phase. Our candidates were PIAS1 and PIAS4, which have been implicated in SUMOylation events at DSB sites (67,78), and CBX4, which was recently reported to mediate CtIP’s role in end resection (46). siRNAs targeting each of the three E3 ligases were transfected into HeLa His_10-_SUMO-2 cells as they were being synchronized to S phase, and His pull-down was performed to enrich SUMO-2-CtIP. While each E3 ligase was knocked down sufficiently, depleting PIAS1 upregulated CtIP expression levels, while depleting PIAS4 and CBX4 downregulated them (Figure 5A, first run). When these fluctuations in basal CtIP expression were accounted for, depleting PIAS4, but not PIAS1 or CBX4, inhibited CtIP SUMOylation in S phase (Figure 5A, second run). Importantly, the effect of PIAS4 knockdown on CtIP SUMOylation was not due to substantial changes in the cell cycle profile (Figure 5B). To complement the depletion experiment, overexpression of FLAG-tagged PIAS4 in the same cells enhanced the abundance of SUMO-2-CtIP, despite even loading of samples into the His pull-down fraction (Figure 5C). Notably, this effect was more pronounced in cells synchronized to S phase relative to asynchronous cells, underscoring the notion that PIAS4 SUMOylates CtIP during S phase. As an E3 SUMO ligase for CtIP, we then predicted that PIAS4 would interact with CtIP, and pursued Co-IP experiments to test this. Indeed, GFP-tagged CtIP was able to co-immunoprecipitate endogenous PIAS4 in U-2 OS cells (Figure 5D). In addition, a U-2 OS cell line stably expressing GFP-CtIP-WT (Supplementary Figure S6B) co-immunoprecipitated more PIAS4 when it had been synchronized to S phase than when it was grown asynchronously (Figure 5E), congruent with the rise in CtIP SUMOylation we observe during S phase (Figure 3D, Supplementary Figure S4C). Intriguingly, inducing replication fork stalling with HU caused a dose-dependent dissociation of PIAS4 from GFP-CtIP (Figure 5F), consistent with the reduction in CtIP SUMOylation seen in the presence of HU and other replication stress agents (Figure 3E-F). All in all, the Co-IP experiments support the notion that PIAS4 associates with CtIP during S phase to promote its SUMOylation. Combining them with the PIAS4 depletion and overexpression experiments, we conclude that PIAS4 is the main E3 ligase for SUMOylating CtIP in S phase.

### K578 is a key CtIP SUMOylation site

**Figure 6.**
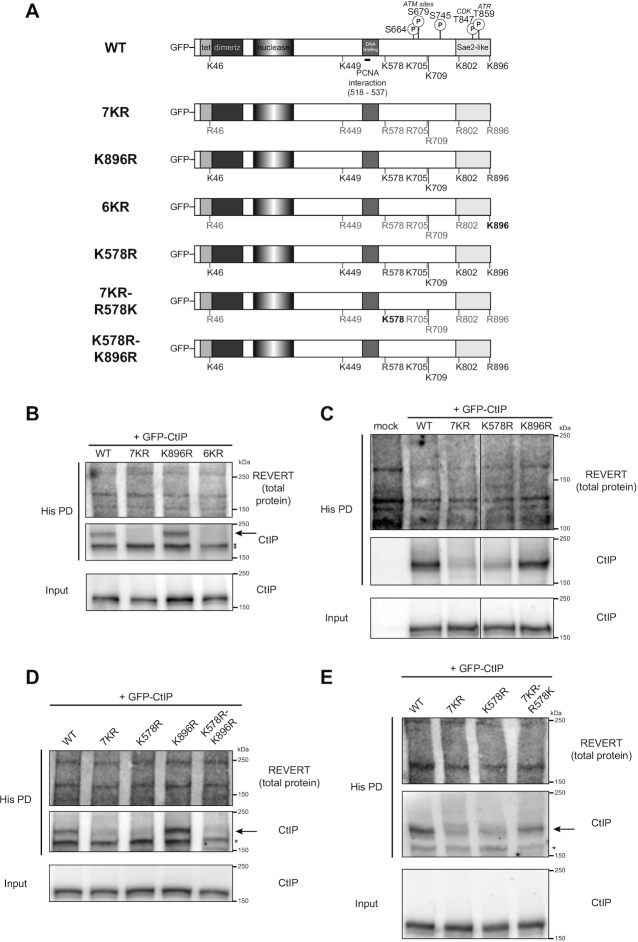
K578 is a key CtIP SUMOylation site. (**A**) Schematic diagrams of CtIP domain structure of wildtype (WT) CtIP and relevant phosphorylation and SUMOylation sites in this study, along with residues substituted in the corresponding site mutants of GFP-tagged CtIP. ‘Tet’: tetramerization domain; ‘dimeriz’: dimerization domain; ‘nuclease’: endonuclease domain, ‘PCNA interaction’: also known as PIP-Box. In (B–E), HeLa His_10_-SUMO-2 cells were synchronized to mid-S phase. 24 h prior to harvest, they were transfected with the indicated GFP-CtIP constructs, then portioned into input control and His pull-down (His PD) fractions and processed accordingly. * indicates an exogenous CtIP immunoreactive band that is not of interest; it may be lower molecular weight SUMO-2-modified CtIP, or unmodified GFP-CtIP retained in the His PD fraction due to high expression. (**B**) Shown is a representative result of three independent experiments. (**C**) The solid line defines where an intervening lane was spliced out of the image. Shown is a representative result of three independent experiments. (**D**) Shown is a representative result of two independent experiments. (**E**) Shown is a representative result of three independent experiments.

Having narrowed down specific factors involved in SUMOylating CtIP in S phase, we wished to determine which particular lysine residue(s) CtIP was SUMOylated on. Recent work from Pablo Huertas’ group uncovered a role for CtIP SUMOylation on residue K896 (46). Using GPS-SUMOsp2.0 software to predict putative SUMOylation sites on CtIP, Soria-Bretones and colleagues selected seven residues as potential SUMO sites: K46, K449, K578, K705, K709, K802 and K896 (Figure 6A) (46). Interestingly, they observed that substituting K896 with the positively-charged but non-SUMOylatable arginine residue yielded functional defects, preventing CtIP’s recruitment to an I-*Sce*I-induced DSB, impairing DNA end resection and RAD51 accumulation, and increasing genomic instability, although having little impact on the CtIP SUMOylation detectable by immunoblot (Supplementary Figure 3G in their report) (46). To further refine which of the six remaining residues selected by Soria-Bretones *et al.* were potential SUMOylation sites, we used an internal deletion panel of FLAG-tagged CtIP constructs, of which the D6 construct described earlier is a part of (47), to assess which regions of CtIP mediated its bulk SUMOylation. Along with D6, the panel consisted of mutants with large deletions that overlap with the remaining sites chosen by Soria-Bretones *et al.*: D3 (residues 369–495 deleted), D4 (496–695 deleted) and D5 (695–778 deleted) (Supplementary Figure S7A). We ignored construct D1 (17–160 deleted) as we did not wish to interfere with CtIP’s N-terminal oligomerization region, shown to be important for CtIP function (47,68,69). Despite variable expression of each construct in HeLa His_10-_SUMO-2 cells, mutants D4 and D6 showed pronounced inhibition of SUMOylation, unlike D3 and D5 (Supplementary Figure S7B). Having already observed an impact of the C-terminus on CtIP SUMOylation via D6 (Figure 4A), the dramatic loss of SUMOylation in D4 suggested residue K578, or potentially other residues within 496–695, could be potential SUMOylation sites or be mediating CtIP SUMOylation.

After obtaining the arginine substitution mutants of GFP-CtIP used by Soria-Bretones *et al.* in their study (46), we proceeded to evaluate which of the seven predicted residues were SUMOylation sites on CtIP. We expressed the mutants in HeLa His_10-_SUMO-2 cells synchronized to S phase, and subjected the cells to His-tag purification. Consistent with Soria-Bretones *et al.* (46), a mutant where all seven predicted sites had been substituted with arginine (7KR) (Figure 6A) strongly reduced the amount of SUMOylated GFP-CtIP enriched in the His pull-down fraction relative to WT-CtIP (Figure 6B), suggesting one or more of the seven predicted residues was responsible for the majority of CtIP SUMOylation. We did not observe a reduction of SUMOylation in the K896 mutant, while the 6KR mutant (where six of the predicted sites except K896 were mutated to arginine) (Figure 6A) sharply diminished CtIP SUMOylation to a level similar to that of 7KR (Figure 6B). This suggests that K896’s contribution to bulk CtIP SUMOylation is very minor, and that one or more of the remaining six sites is instead responsible. With residues K449, K705, K709 located within internal deletions in mutants that did not reduce CtIP SUMOylation (D3 and D5), and K578 located within the deleted residues of mutant D4, which showed remarkable loss of SUMOylation (Supplementary Figure S7B), we moved to investigate if K578 specifically was a CtIP SUMOylation site.

The K578R mutant of GFP-CtIP was generated by site-directed mutagenesis and transfected into S phase-synchronized HeLa His_10-_SUMO-2 cells. Relative to GFP-CtIP-WT, K578R abolished SUMOylation to an extent similar to the 7KR mutant (Figure 6C), suggesting K578 is a major site responsible for CtIP SUMOylation. In addition, unlike the K896R single mutant, a double mutant where both K578 and K896 were mutated to arginine (K578R-K896R) (Figure 6A) exhibited a similar reduction in SUMOylation as both the K578R and 7KR mutants (Figure 6D), emphasizing the key contribution of K578 to bulk CtIP SUMOylation. To determine if K578 alone was responsible for bulk SUMOylation, or if the remaining predicted sites contributed, we generated a mutant that would allow only K578 to be SUMOylatable out of the seven predicted sites. The mutant, 7KR-R578K, was prepared by reverting residue R578 to lysine in the 7KR construct (Figure 6A). Upon expression in S phase or asynchronous HeLa His_10-_SUMO-2 cells, the reversion partially restored CtIP SUMOylation, but not to the extent of WT-CtIP (Figure 6E, Supplementary Figure S7C). This confirmed that K578 was a SUMOylatable residue, while suggesting its SUMOylation is a pre-requisite for SUMOylation events on the other six residues to constitute the rest of CtIP SUMOylation. To recapitulate the effect of K578R in a construct that did not utilize a bulky GFP tag, we incorporated the mutation into HA-tagged CtIP, observing yet again a substantial loss in SUMO-2-modified CtIP in the presence of K578R, even in cells that were asynchronous (Supplementary Figure S7D). Finally, we sought to demonstrate the importance of K578 in CtIP SUMOylation by aligning amino acid sequences of CtIP orthologues using Clustal Omega. Consistently, K578, amid the canonical ψ-K-x-E SUMOylation motif (79–81) (with ψ representing a bulky, hydrophobic amino acid), is conserved among mammalian orthologues of CtIP as well as in chicken (Supplementary Figure S7E). Taken together, our data uncover a novel SUMOylation site for CtIP at residue K578. Moreover, they reveal a dynamic interplay between SUMO sites, with K578 SUMOylation serving as a prerequisite for SUMOylation on other residues, a role not detectably manifested by K896.

### Cells expressing K578R mutant CtIP show defects in DNA end resection and homologous recombination

**Figure 7.**
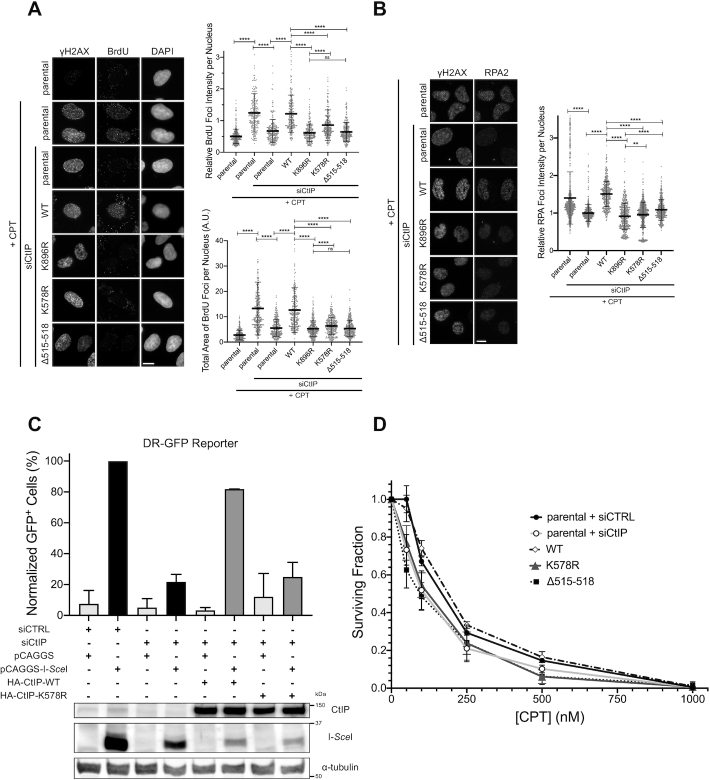
Cells expressing K578R mutant CtIP show defects in DNA end resection and homologous recombination. (**A**) Left panel: parental U-2 OS cells or U-2 OS stably expressing the indicated siRNA-resistant GFP-CtIP constructs were transfected with siRNA to CtIP (or not) and treated with 1 μM CPT for 1 h (or not), then processed for IF staining. Cells were cultured in BrdU-containing media prior to treatment with CPT. Cells sensitive to CPT (seen by the induction of γH2AX foci, with the exception of the untreated condition) were quantified for BrdU foci (right panels). IF micrographs are representative of three independent experiments; 249–299 cells per condition from two independent experiments (total foci intensity) and 299–340 cells per condition from three independent experiments (total foci area) were quantified. (**B**) Similar to (A) but without BrdU in the culture media, and IF staining for RPA2 instead of BrdU. Micrographs are representative of four independent experiments; 467–748 cells per condition from three to four independent experiments were quantified for total RPA2 foci intensity. Asterisks depict statistically significant differences as determined by a two-tailed, unpaired, non-parametric Student's *t*-test (Mann–Whitney): ns (not significant), ** (*P*< 0.01), **** (*P*< 0.0001). (**C**) DR-GFP homologous recombination reporter assay in U-2 OS stably expressing the DR-GFP cassette. The cells were transfected with non-targeting (siCTRL) or CtIP-targeting (siCtIP) siRNA. 24 h later, they were transfected with the same siRNAs, and either pCAGGS empty vector or pCAGGS-I-*Sce*I, in combination with siRNA-resistant constructs encoding HA-CtIP-WT or -K578R or not. GFP^+^ cells were assessed via flow cytometry 24 h later. Data presented are the means of two independent experiments (top panel). An immunoblot confirming the transfection combinations for one experiment is presented beneath (bottom panel). (**D**) Clonogenic survival assay of parental U-2 OS transfected with siCTRL or siCtIP, or U-2 OS cell lines stably expressing GFP-CtIP-WT, -K578R or Δ515–518 and transfected with siCtIP. Cells were treated with CPT at the indicated concentrations for 1 h, and colonies were allowed to form over ∼10 days. Survival data is presented as mean ± standard deviation from three independent experiments.

We next sought to study the functional effects of CtIP reduced in SUMOylation using the mutants K578R and Δ515–518 (mutant with attenuated PCNA interaction, Figure 4G). We first asked if cells expressing these mutants exhibited a defect in DNA end resection. To do this, we generated U-2 OS cell lines stably expressing siRNA-resistant GFP-CtIP-WT, -K578R, -K896R and -Δ515–518 (Supplementary Figure S6B). The cells were then depleted of endogenous CtIP with siRNA, and treated with CPT to induce end resection, which we measured by quantifying native BrdU (Figure 7A) and RPA (Figure 7B) foci in γH2AX^+^ cells. Parental U-2 OS cells were capable of forming native BrdU and RPA foci in response to CPT, but this was severely impaired when CtIP was depleted. Expressing WT-CtIP could restore these foci to similar levels as parental cells, but adding back K578R or Δ515–518 could not, or caused only a slight rescue of foci formation, indicating both mutants were defective in promoting end resection. Critically, the K896R mutant also could not recover RPA and BrdU foci to levels in cells expressing WT-CtIP, in line with the findings of Soria-Bretones *et al.* (46).

We then explored mechanistically how K578R and Δ515–518 are unable to promote end resection. We first asked if both mutants were capable of being recruited to sites of DNA damage generated by laser microirradiation. GFP-CtIP-K578R was recruited and retained on laser tracks efficiently, with similar kinetics as GFP-CtIP-WT (Supplementary Figure S8A, B), indicating impaired recruitment was not a factor in its inability to promote end resection, and that SUMOylation on K578 is not required for CtIP’s recruitment to sites of damage. On the other hand, the Δ515–518 mutant recruited poorly to laser-damaged DNA, providing an explanation for its inability to stimulate resection. We thus continued investigating functional impacts of the K578R mutation, hypothesizing that SUMOylation at K578 could be promoting CtIP’s known interactions with the MRN complex and BRCA1. By Co-IP, the K578R mutant co-immunoprecipitated all the aforementioned proteins as efficiently as WT-CtIP (Supplementary Figure S8C), implying CtIP’s association with these proteins is not dependent on the K578 residue being a functional modification site. Seeing no alteration in recruitment or protein interactions, we resorted to reconstituting end resection *in vitro* to see if K578R-CtIP was impaired in its ability to stimulate the MRN complex in cleaving streptavidin-blocked double-stranded DNA (6). We successfully expressed and purified phosphorylated WT- and K578R-CtIP and the MRN complex in *Sf*9 cells (Supplementary Figure S8D, E). To our surprise, both phosphorylated WT- and K578R-CtIP were capable of stimulating MRN endonuclease activity, and to a similar extent (Supplementary Figure S8F). However, as we could not detect higher molecular weight forms of purified CtIP indicative of SUMOylation (Supplementary Figure S8D), it is likely that most of the purified CtIP was not SUMOylated and that a difference in SUMOylation status did not exist between WT- and K578R-CtIP. Nevertheless, our *in vitro* data demonstrates that the K578R mutant is inherently as capable of stimulating MRN activity as WT-CtIP, therefore its defect in promoting end resection *in vivo* is not due to a change in catalytic activity arising from the arginine substitution. This suggests that for WT-CtIP in the cell, SUMO modification at K578 may promote conformational changes that alter MRN activity, or alter CtIP’s interactions with other proteins. Perhaps it is these changes that fully activate CtIP’s ability to stimulate MRN-dependent resection, and these changes are lost when the K578 site is not modifiable.

We next wondered if there were further functional impacts from the loss in end resection in cells expressing K578R-CtIP. As end resection is an initiating step in homologous recombination (3), we predicted that the process of HR would be disrupted if end resection was impaired. To test this, we performed the DR-GFP HR reporter assay (Supplementary Figure S1A) in cells depleted of endogenous CtIP and complemented with either HA-tagged WT- or K578R- siRNA-resistant CtIP (Figure 7C, Supplementary Figure S7D). As expected, the frequency of HR events was dramatically decreased in cells depleted of CtIP. Fittingly, expressing HA-CtIP-WT was able to rescue HR almost to levels seen in cells treated with non-targeting siRNA, while expressing HA-CtIP-K578R was strikingly unable to, demonstrating the significance of CtIP SUMOylation at K578 on proper HR function. Taking this further, we anticipated that cells expressing K578R-CtIP, with their diminished end resection and consequently HR capacities, would be more sensitive to DNA damage by DSBs. To address this, we performed a clonogenic survival assay on CtIP-depleted stable cell lines expressing GFP-CtIP-WT or -K578R and challenged with CPT (Figure 7D, Supplementary Figure S6B). Accordingly, unlike WT-CtIP expressing cells, those expressing the CtIP-K578R mutant were as sensitive to CPT as parental U-2 OS cells depleted of CtIP with siRNA. This sensitivity was also seen for the Δ515–518 mutant, which is impaired in recruitment to sites of DNA damage, SUMOylation and thus DNA end resection (Supplementary Figure S8A, B; Figures 4H, 7A, B). To summarize, modification of CtIP at residue K578 by SUMOylation promotes DNA end resection, and substituting K578 with arginine results in impaired end resection and HR activity and poorer survival in response to CPT.

### K578 is not required for CtIP recruitment during replication stress or its interaction with PCNA

The fact that CtIP SUMOylation decreases in the presence of HU, aphidicolin and low dose CPT (Figure 3E, F) suggests SUMO-2 modification may mediate CtIP’s functions in the response to replication stress. We thus performed experiments to explore the functional consequences of an inability to SUMOylate CtIP in the context of replication stress. We previously found that CtIP is recruited to sites of replication stress, seen by its ability to form foci in response to HU and low dose CPT (Supplementary Figure S2A). We then asked how a loss of SUMOylation at K578 would impact CtIP recruitment to stalled replication forks. We depleted endogenous CtIP in U-2 OS cells stably expressing GFP-CtIP-WT or -K578R, then treated them with HU and evaluated their abilities to form foci. U-2 OS stably expressing the PCNA interaction mutant GFP-CtIP-Δ515–518 (Figure 4G) were also used in these experiments, as previous observations showed the interaction with PCNA recruits CtIP to active replication centers (25). In response to HU, cells expressing K578R-CtIP exhibited a partial reduction in foci-forming ability compared to WT-CtIP, as opposed to those expressing Δ515–518, which were severely inhibited (Supplementary Figure S9A). While this indicates an interaction with PCNA is needed for CtIP recruitment to sites of replication stress, in line with the literature (25), it is clear that unlike the Δ515–518 mutant, SUMOylation of CtIP at K578 is dispensable for recruitment to these sites.

Earlier, we also observed that the interaction with PCNA promotes CtIP SUMOylation (Figure 4G, H). We next pursued the converse question: if SUMOylation at K578R impacts CtIP’s interaction with PCNA, both under normal and replication stress conditions. We performed co-immunoprecipitations in U-2 OS cells depleted of endogenous CtIP and transfected with RFP-PCNA and GFP-CtIP-WT or -K578R. Both the forward and reverse Co-IPs did not show a reduction in the CtIP-PCNA interaction for K578R-CtIP relative to WT at the steady state, unlike the Δ515–518 mutant (Supplementary Figure S9B-D). Moreover, the interaction of GFP-CtIP and endogenous PCNA was not altered in the presence of replication stress induced by HU, whether WT- or K578R-CtIP was immunoprecipitated as the bait (Supplementary Figure S9E). Thus, we conclude residue K578 is not required for the interaction between CtIP and PCNA.

### K578R expression phenocopies a fork protection defect in CtIP-depleted cells during replication stress

**Figure 8.**
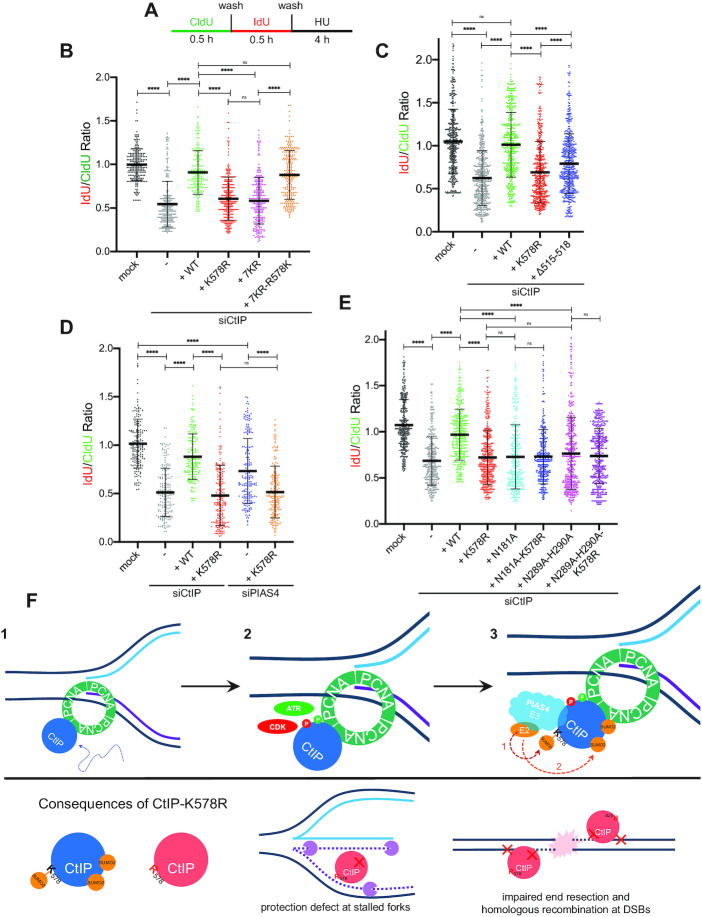
K578R expression phenocopies a fork protection defect in CtIP-depleted cells during replication stress. (**A**) Labeling and 2 mM HU treatment protocol for all DNA fiber experiments performed. (B–E) IdU/CldU ratio scatterplots of spread DNA fibers from U-2 OS cells transfected with siRNA targeting CtIP or PIAS4 or mock transfected ∼48 h prior to labeling, transfected with the indicated GFP-CtIP constructs or not (–) ∼16 h prior to labeling, then treated according to (A). The mean and standard deviation are displayed. Asterisks depict statistically significant differences as determined by a two-tailed, unpaired, non-parametric Student's *t*-test (Mann–Whitney): ns (not significant), **** (*P*< 0.0001). (**B**) 182–290 fibers per condition were sourced from two independent experiments. (**C**) 476–520 fibers per condition were sourced from two independent experiments. (**D**) 169–204 fibers per condition were sourced from two independent experiments. (**E**) 332–423 fibers per condition were sourced from two independent experiments. (**F**) Schematic diagram of the model of the data. See text for details.

A recent study demonstrated that CtIP protects stalled replication forks from degradation (26). Here, the presence of CtIP limited stalled replication forks from over-resection by DNA2 exonuclease and prevented genomic instability, virtues lost when mutations that abrogate CtIP’s apparent intrinsic flap endonuclease activity were introduced (26,82,83). We therefore sought to determine if SUMOylation of CtIP at K578 could play a role in replication fork protection, despite the K578R mutant still being able to recruit to replication foci (Supplementary Figure S9A). To directly visualize the impact of K578R substitution on fork dynamics, we performed the DNA fiber spreading assay. Briefly, we sequentially pulse-labeled newly synthesized DNA using two halogenated nucleosides (5-chloro-2′-deoxyuridine (CldU) first, then 5-iodo-2′-deoxyuridine (IdU) second), then treated with HU to stall replication fork progression and potentially initiate nascent strand degradation (Figure 8A; the typical appearance of spread fibers is shown in Supplementary Figure S10A). The ratio of the resulting IdU and CldU tract lengths in the spread DNA fibers was then used as a readout for replication fork protection; IdU/CldU ratios near 1 reflected little or no degradation (fork protection), whereas ratios <1 indicated degradation of newly synthesized DNA. The assay was performed on U-2 OS cells depleted of endogenous CtIP and complemented with WT- or mutant GFP-CtIP. Consequently, depleting CtIP consistently reduced the IdU/CldU ratio from ∼1 to 0.5–0.7, and the ratio was restored to 0.91 when GFP-CtIP-WT was added back (Figure 8B), confirming the reported loss of fork protection in the absence of CtIP (26). Interestingly, expressing the 7KR mutant could not restore fork protection, nor could K578R-CtIP. However, the 7KR-R578K mutant recovered the IdU/CldU ratio to near WT levels (Figure 8B). This implicates K578 as the residue among the seven predicted SUMOylation sites that mediates CtIP’s role in replication fork protection. In support of a role for CtIP SUMOylation in fork protection, the Δ515–518 mutant, which interacts less with PCNA and is inhibited in SUMOylation (Figure 4G, H), reduced the IdU/CldU ratio in a similar manner to CtIP-K578R (Figure 8C), although the value was slightly higher, perhaps because K578 was still intact as a SUMOylation site. Secondly, depleting SUMO E2 UBC9 also produced the nascent DNA degradation phenotype (Supplementary Figure S10B), suggesting SUMOylation events in general promote fork protection. Thirdly, as PIAS4 SUMOylates CtIP in S phase (Figure 5), we predicted that reducing PIAS4 levels would lead to more nascent DNA degradation. Certainly, knockdown of PIAS4 reduced the IdU/CldU ratio relative to mock transfected cells, but not to the extent of cells expressing the K578R mutant (Figure 8D), suggesting fork protection overall is mediated by PIAS4 and other E3 SUMO ligases. Critically, however, depleting PIAS4 while expressing K578R-CtIP resulted in an IdU/CldU ratio similar to expressing the K578R mutant alone (Figure 8D), demonstrating that CtIP modification at K578 and PIAS4 activity reside within the same pathway and are epistatic. Together, our DNA fiber data demonstrate that an inability to SUMOylate CtIP results in defective fork protection in response to HU, with the CtIP-K578R mutant phenocopying the fork over-resection defect seen by the loss of CtIP.

Since the purported flap endonuclease activity of CtIP (82,83) was reported to mediate CtIP’s role in replication fork protection (26), we were curious if SUMOylation at K578 controls this activity. The apparent endonuclease activity can be inhibited by loss of function mutations at residues N181 or N289 and H290 (82,83). We thus generated both mutants in GFP-CtIP by site-directed mutagenesis, along with double mutants combining ‘nuclease deficiency’ with K578R (N181A-K578R, N289A-H290A-K578R). As a control, we found that neither of the solely ‘nuclease-deficient’ mutants were impacted in the ability to be SUMOylated during S phase in HeLa His_10-_SUMO-2 cells, unlike K578R (Supplementary Figure S10C). Only the ‘nuclease-deficient’-K578R double mutants were strongly reduced in CtIP SUMOylation, and to the same level as K578R alone (Supplementary Figure S10C), indicating that ablation of K578 was responsible for the reduction in SUMOylation. Intriguingly, K578R, both ‘nuclease-deficient’ mutants, and the ‘nuclease-deficient’-K578R combination mutants all showed similar IdU/CldU ratios (Figure 8E). This suggests that both SUMOylation at K578 and the functions of residues N181 and N289/H290 are within the same pathway. While this implies K578 SUMOylation could control CtIP’s purported endonuclease activity, we could not test the idea, as we were unable to detect any flap endonuclease activity in purified CtIP to start (Supplementary Figure S10D). Nevertheless, this experiment shows K578 SUMOylation is epistatic with whatever functions residues N181 and N289/H290 mediate, and our evidence overall implicates K578 SUMOylation in the mechanism by which CtIP protects stalled replication forks from over-resection.

## DISCUSSION

In this study, we sought to find potential SUMO targets in the HR pathway and characterize how SUMOylation could affect their function. Use of the SUMOylation inhibitor GA suggested CtIP was a substrate for SUMOylation, as its recruitment to sites of DSBs was impaired upon GA treatment, along with the process of DNA end resection, which CtIP is known to promote (5–7) (Figure 1G-I, Supplementary Figure S1D). By nickel affinity purification of His_10_-SUMO-2ylated proteins, we later detected DSB damage-independent (Figure 3A, B, Supplementary Figure S4D) and constitutive (Figure 2B–D) CtIP SUMOylation *in vivo*. In line with this, a recent report by Soria-Bretones *et al.* also found CtIP to be constitutively SUMOylated, and at similar levels with or without DNA damage (46). Soria-Bretones and coworkers showed that this was mediated by the E3 SUMO ligase CBX4, which when depleted inhibited DNA end resection (46). Furthermore, they found that SUMOylation at the near-C-terminal residue K896 was required for proficient DNA end resection, RAD51 foci formation, maintenance of genomic stability and CtIP’s recruitment to I-*Sce*I-generated DSBs, as these were all inhibited in cells expressing the K896R mutant but could be rescued when SUMO-1 was fused to the C-terminus of CtIP (46). In our study, we establish a cell cycle dependency for CtIP SUMOylation, with the modification strikingly stimulated during S phase (Figure 3D and Supplementary Figure S4C). We then uncovered a SUMOylation site on CtIP, K578, that sits within a canonical ψ-K-x-E SUMOylation motif (Supplementary Figure S7E). Comparing our findings, we and Soria-Bretones *et al.* show that constitutive CtIP SUMOylation at residues K578 or K896 (46) is important for DNA end resection, and to similar extents (Figure 7A, B). It might be that both sites are functionally critical to promote end resection, thus end resection is blocked whenever one or the other site is ablated. More enticingly, however, it could be that K896 SUMOylation controls end resection, but that K578 SUMOylation is a prerequisite for K896 SUMOylation (see below). Meanwhile, our studies do report contrasting results for the dependence of SUMOylation on CDK phosphorylation at T847, where we find T847 phosphorylation does partially mediate SUMOylation (Figure 4C). Additionally, CtIP SUMOylation was promoted by the E3 ligase PIAS4, not CBX4, in our study (Figure 5A, C). These two contradictions could be a result of our emphasis on S phase-synchronized cells and the SUMO isoform SUMO-2. We reason that PIAS4 SUMOylates CtIP in S phase, and speculate additional SUMOylation by CBX4 could then prepare CtIP to function in DNA end resection.

CtIP’s function in end resection is tightly governed by multiple PTMs, including phosphorylation (35–37), ubiquitylation (32) and SUMOylation (46). For instance, CDK phosphorylation of T847 (and its equivalent in the *S. cerevisiae* orthologue) restricts end resection activity to the S and G_2_ cell cycle stages (35,84). Furthermore, ATR phosphorylation at T859 enables CtIP recruitment to DNA to activate end resection (36). Our work reveals a dynamic interplay between the PTMs on CtIP. Firstly, our data indicates CDK- and ATR-mediated phosphorylations promote CtIP SUMOylation during S phase (Figure 4B–E), suggesting phosphorylation events are upstream of CtIP SUMOylation. In support of this, both kinase activities were linked for CtIP phosphorylation in *Xenopus* oocytes, where phosphorylation at the residue corresponding to CDK site T847 preceded ATR phosphorylation at the equivalent of T859 (36). In addition, a connection between CDK activity and human CtIP SUMOylation was found in a mass spectrometry screen, where peptides of K578-SUMOylated CtIP were co-modified with CDK-dependent phosphorylation, and CtIP SUMOylation at different sites was altered in the presence of a CDK inhibitor (85). Secondly, we uncover mechanistic intricacies in CtIP SUMOylation. Unlike the K896R mutation, the K578R substitution dramatically reduces CtIP SUMOylation (Figure 6C–E, Supplementary Figure S7C, D), while combining both mutations in the K578R-K896R double mutant reduces CtIP SUMOylation to a level similar to K578R alone (Figure 6A, D). This suggests SUMOylation on K896 is likely a low abundance modification, perhaps only for the fraction of CtIP engaged in end resection (46), and is consistent with Soria-Bretones *et al.* who found the K896R mutant exhibited similar SUMOylation levels as WT-CtIP (46). Astonishingly, we found that K578 contributes prominently to CtIP SUMOylation not only as a SUMOylation site, but by priming SUMOylation *en masse* at other residues within CtIP when it is SUMOylated. This was inferred from the observation that the K578R single mutant reduces SUMOylation levels similar to that of the 7KR mutant (where all seven potential SUMOylation sites are blocked), yet the 7KR-R578K mutant (where of the seven blocked SUMOylation sites, only K578 was restored) could only partially rescue SUMOylation levels to that of WT-CtIP (Figure 6A, E, Supplementary Figure S7C). Thus it appears at least some of the other six putative sites contribute to CtIP SUMOylation, but in a manner dependent on the SUMOylation of K578. It may be that SUMOylation at K578 is a prerequisite for activatory conformational changes in CtIP that then allow other lysine residues to become accessible for SUMOylation.

While HR proteins are involved in fork reversal and restart (16–22), their precise roles and regulation at replication forks are poorly understood compared to their functions at DSBs. Our data fosters three implications for CtIP in fork biology. Firstly, our findings illustrate the importance of CtIP’s interaction with PCNA (25). We demonstrate that disrupting the interaction via Δ515–518 (Figure 4G, Supplementary Figure S9D) prevents CtIP recruitment to sites of DNA damage as well as replication stress foci (Supplementary Figures S8A, B, S9A). Interestingly, while CtIP-Δ515–518 exhibits a recruitment defect, this is not manifested in the K578R mutant (Supplementary Figures S8A, B, S9A), implying that the inhibition of CtIP recruitment upon GA treatment (Figure 1I, Supplementary Figure S2B) may depend on SUMOylation on other sites of the protein, or perhaps multiple other SUMOylation events beyond those on CtIP. That CtIP-K578R is markedly less SUMOylated than WT, beyond the contribution of the K578 site alone (Figure 6C–E, Supplementary Figure S7C, D), but still is recruited efficiently also suggests complete SUMOylation is not required for CtIP accumulation at sites of replication stress or DNA damage. This supports the notion that CtIP SUMOylation occurs downstream of its accrual on chromatin. We propose then that the PCNA-CtIP interaction targets CtIP to actively replicating DNA for rapid responses to DNA damage and replication stress. This could then allow CtIP to be SUMOylated (Figure 4H), which we find is critical for its functions in DNA end resection and fork protection (Figures 7A, B, 8B–D). While a portion of the Δ515–518 mutant is still chromatin-enriched (Supplementary Figure S6B), perhaps localizing CtIP to PCNA brings CtIP in the vicinity of PIAS4 for its SUMOylation, in line with a report that PCNA can be SUMOylated by PIAS4 (86). Second of all, our data demonstrate that SUMOylation of K578 is necessary and sufficient for protecting stalled replication forks from excessive nucleolytic degradation (Figure 8). This is made evident with the 7KR-R578K (Figure 8B) mutant being proficient in fork protection, despite ablation of the remaining six potential SUMOylation sites (Figure 6A). While K578R and point mutants causing CtIP to be ‘nuclease-deficient’ (82,83) were epistatic in the fork degradation phenotype (Figure 8E), we were unable to detect this intrinsic endonuclease activity in purified CtIP (Supplementary Figure S10D) and thus could not determine if SUMOylation regulated the activity. Other groups have not been able to detect nuclease activity in CtIP or its orthologues (6,7,69). We therefore speculate SUMOylation triggers conformational changes that control CtIP’s function as a co-factor for an associated endonuclease activity. This activity promotes fork protection and is somehow mediated by residues N181 and N289/H290 within CtIP, perhaps by facilitating protein-protein interactions. Thirdly, the increase in CtIP SUMOylation during S phase (Figures 3D, 4B, D, and Supplementary Figure S4C, D) raises the intriguing possibility that CtIP could play a role in DNA replication. This is bolstered by evidence that CtIP interacts constitutively with the processivity factor PCNA (25), an observation we have reproduced (Figure 4F, Supplementary Figure S6A), forms foci at sites of active DNA replication (25) and during replication stress (Supplementary Figure S2A), is recruited to ongoing replication forks as detected by iPOND (isolation of proteins on nascent DNA) (24), and that SUMOylated CtIP is enriched on chromatin (Figure 2D) and is promoted by CDK activity (Figure 4B, C). Notably, we find that replication stress inducing agents reduce CtIP SUMOylation (Figure 3E, F), and fittingly HU treatment causes the dissociation of the E3 SUMO ligase PIAS4 from CtIP (Figure 5F). Consistent with this, a mass spectrometry study detected K578 as a SUMOylation site on CtIP, and classified it as a dynamically regulated site, being markedly deSUMOylated in response to HU treatment at 2 and 24 h (65). Thus, it appears CtIP is SUMOylated during fork progression, but this is reduced during prolonged fork stalling. Perhaps these changes in SUMOylation level switch CtIP between different roles depending on the conditions at the fork. Interestingly, while we observe a reduction in CtIP SUMOylation in response to HU (Figure 3F), the SUMOylation-deficient CtIP-K578R mutant still exhibits a fork protection defect (Figure 8B-E). This suggests CtIP must first be SUMOylated in S phase, then deSUMOylated during prolonged fork stalling, in order to protect halted forks from nascent DNA strand degradation.

Taken together, our data supports the following model (Figure 8F). CtIP is recruited to chromatin by an interaction with PCNA, which facilitates its targeting to active DNA replication foci during S phase. CDK-dependent phosphorylation events (at T847 and other residues), along with ATR-dependent phosphorylation (at sites such as S664, S679 and/or S745), predispose CtIP for constitutive modification by SUMO-2 during S phase. This SUMOylation is facilitated by the E3 SUMO ligase PIAS4. SUMOylation on residue K578 then licenses CtIP to be SUMOylated on other sites. SUMOylated CtIP is an activated version of the protein that promotes DNA end resection, and subsequently HR, and prevents over-resection of newly synthesized DNA at stalled replication forks, functions that are disrupted when K578 is not modifiable by SUMOylation (Figure 8F). Overall, our data provide a link between CtIP SUMOylation and fork protection (26). Our work expands the functions of SUMOylation in regulating CtIP beyond its recruitment to DSBs, promotion of end resection (46), and solubility (87), and validates K578 as a *bona fide* SUMOylation site with functional impacts.

## Supplementary Material

gkaa1232_Supplemental_File
