# Supplementary material for: SUMOylation mediates CtIP’s functions in DNA end resection and replication fork protection
Source: Nucleic Acids Res. 2021 Jan 6;49(2):928–53. doi: 10.1093/nar/gkaa1232 (PMC7826263; doi:10.1093/nar/gkaa1232)
Supplement: gkaa1232_Supplemental_File [file gkaa1232_supplemental_file.pdf]

SUPPLEMENTAL INFORMATION

SUMOylation Mediates CtIP's Functions in DNA End Resection and Replication Fork Protection

Andrew J. Locke[1], Lazina Hossain[1†], Glynnis McCrostie[1†], Daryl A. Ronato[2,3], Amira Fitieh[1,4], Tanzeem Ahmed Rafique[1], Fatemeh Mashayekhi[1], Mobina Motamedi[1], Jean-Yves Masson[2,3], and Ismail Hassan Ismail[1,4*]

[1] Division of Experimental Oncology, Department of Oncology, Faculty of Medicine & Dentistry, University of Alberta; Cross Cancer Institute, Edmonton, Alberta, T6G 1Z2, Canada

[2] Oncology Division, CHU de Québec-Université Laval Research Center, Québec City, Québec, G1R 3S3, Canada

[3] Department of Molecular Biology, Medical Biochemistry and Pathology, Faculty of Medicine; Laval University Cancer Research Center, Université Laval, Québec City, Québec, G1V 0A6, Canada

[4] Biophysics Department, Faculty of Science, Cairo University, Giza, Egypt

[†] These authors contributed equally to this work.

SUPPLEMENTAL FIGURE LEGENDS

Supplemental Figure S1: Related to Figure 1

A) Schematic representation of the DR-GFP homologous recombination reporter cassette (1). The reporter contains two copies of a modified GFP gene, both of which are mutated to produce non-fluorescent protein products. One incorporates an I-*Sce*I cutting site containing two stop codons (*SceGFP*); the other encodes an internal fragment of GFP (*iGFP*). Expression of I-*Sce*I endonuclease generates a double-strand break in *SceGFP*. Repair of the break by gene conversion produces functional GFP. **B)** Immunoblot showing reduction in UBC9 expression 24 hours after transfecting GFP-tagged Gam1 into HeLa cells, representative of 2 independent experiments. **C)** HeLa cells were transfected with GFP-Gam1 or mock transfected. The cells were harvested 24 hours later and processed for DNA content analysis. Shown are the averages of 3 independent experiments. **D)** Quantitation of BrdU foci intensity in U-2 OS cells cultured in BrdU-containing media, pre-treated for 1 hour with 12 μM GA or 0.025% DMSO, and stimulated for 1 hour in 1 μM CPT in their presence. Evenly scaled IF images of both conditions were compared in tandem, and all γH2AX$^+$ cells were scored for their relative BrdU foci intensity (bright, dim, or no foci). The proportions of cells in each category were calculated per field of view. 40 fields of view pooled from 4 independent experiments were scored per condition. **E) and F)** IF micrographs of U-2 OS cells transfected or not with GFP-Gam1 for 24 hours, after which 1 μM CPT was added for 1 hour. As only cells in S phase are sensitive to CPT, cells that did not respond to CPT are also presented. In **F)**, cells were cultured in BrdU-containing media prior to treatment. Shown are representative images from 3 **(E)** and 2 **(F)** independent experiments. **G) and H)** IF micrographs of U-2 OS cells stably expressing MRE11-GFP **(G)** or plain U-2 OS cells **(H)** pre-treated with 12 μM GA or 0.025% DMSO for 1 hour, subjected to 10 Gy of IR or not, and

recovered for 2 hours in the presence of GA or DMSO. Left panel: representative images from at least 6 independent experiments. Center and right panels: quantifications of total foci intensity (pooled from 3 independent experiments; ≥175 cells per condition for MRE11-GFP, ≥153 cells per condition for NBS1) and foci count (1 experiment; ≥114 cells per condition for MRE11-GFP, ≥103 cells per condition for NBS1). In **G)**, MRE11-GFP foci were quantified from MRE11-GFP foci-positive cells, whereas in **H)**, NBS1 foci were quantified from all cells.

Supplemental Figure S2: Related to Figure 1

A) U-2 OS cells stably expressing GFP-CtIP were treated with the indicated concentrations of CPT or HU for 4 hours and processed for IF staining. Shown are representative images from 2 independent experiments, and GFP-CtIP foci quantifications from 70 – 186 $\gamma H2AX^+$ cells per condition from 1 experiment. All images were scaled evenly for brightness and contrast. **B)** U-2 OS cells stably expressing GFP-CtIP were pre-treated for 30 minutes with 10 μM GA, then treated for 4 hours with 50 nM CPT or 2 mM HU in the presence of GA and processed for IF staining. Shown are representative images from 2 independent experiments, and GFP-CtIP foci quantifications from 75 – 123 $\gamma H2AX^+$ cells per condition from 1 experiment. All images were scaled evenly for brightness and contrast.

Supplemental Figure S3: Related to Figure 2

A) Immunoblot verifying the expression of His_{10}-SUMO-2 in HeLa His_{10}-SUMO-2 cells. **B)** HeLa cells expressing 10XHis-tagged SUMO-2 (His_{10}-SUMO-2) or HeLa cells they were derived from (parent) were portioned into input and His PD fractions and processed accordingly. The His PD fraction was processed using buffers containing 20 mM imidazole, and the fraction loaded

represents 12X more of the starting amount of sample relative to the input. Shown is a representative result of 3 independent experiments. Corresponds to **Fig. 2B**. **C)** HeLa His_{10}-SUMO-2 cells were treated with GA at the indicated concentrations for 2 hours, then portioned into input and His PD fractions and processed accordingly before SDS-PAGE and immunoblotting. Shown is a representative result of 3 independent experiments. **D)** Lysates from HeLa His_{10}-SUMO-2 cells expressing FLAG-tagged wildtype CtIP (WT) or a C-terminal truncation mutant (D6) (see **Fig. S7A**) were blotted for CtIP or FLAG to demonstrate the specificity of the CtIP antibody for the CtIP C-terminus. Shown is a representative result of 3 independent experiments.

Supplemental Figure S4: Related to Figure 3

For **A), C), and D)**, HeLa His_{10}-SUMO-2 cells were treated as indicated, portioned into input control and His PD fractions, processed accordingly for whole cell lysis or Ni-NTA affinity purification, then resolved by SDS-PAGE. **A)** Cells were subjected to 10 Gy of IR or not and allowed to recover for 1 hour. Corresponds to **Fig. 3A**. **B)** HeLa His_{10}-SUMO-2 cells were synchronized by double thymidine block and released for either 3 (mid-S phase), 7 (early G_2 phase), or 11 hours (G_1 phase). The resulting cell lysates were blotted for the expression of His_{10}-SUMO-2 and cell cycle markers (Geminin, for S phase; Cyclin A, for G_2 phase; Cyclin D1, for G_1 phase) (left panel). Right panel: the corresponding cell cycle profiles by DNA content analysis. **C)** HeLa His_{10}-SUMO-2 cells were synchronized by double thymidine block and released for various timepoints to reach different cell cycle phases (0 h for the G_1/S transition, 3 h for mid-S phase, 6 h for S and G_2 phases, 11 h for G_1 phase). **D)** Cells were left asynchronous or synchronized by double thymidine block with release for 2 (mid-S phase) or 12 hours (G_1 phase), after which they

were subjected to 10 Gy of IR or not and allowed to recover for 1 hour. Shown is a representative result of 2 independent experiments.

Supplemental Figure S5: Related to Figure 4

A) Schematic diagrams of the domain structure of GFP-CtIP-WT and the internal deletion mutant -Δ515-518 along with relevant phosphorylation sites. “Tet”: tetramerization domain; “dimeriz”: dimerization domain; “nuclease”: endonuclease domain, “PCNA interaction”: also known as PIP-Box. **B)** Cells were treated as in **Fig. 4B** and processed for DNA content analysis. Shown are the means of 2 independent experiments. **C)** Lysates of HeLa His_{10}-SUMO-2 cells treated for 2.5 hours in 0.1% DMSO, 25 μM roscovitine, 2.5 μM AZD5438, or 10 μM RO-3306 were blotted for the phosphorylation of CDK substrates. Ser2 in the heptapeptide repeats of the RNA polymerase II subunit Rpb1 C-terminal domain (CTD) is phosphorylated by CDK9 (2), which is a target of roscovitine and AZD5438 (3, 4). Thr320 of protein phosphatase 1 alpha (PP1α) is phosphorylated by CDK1 (5), which is a target of AZD5438 and the target for RO-3306 (4, 6). **D)** Immunoblot of input and His PD fractions of HeLa His_{10}-SUMO-2 cells transfected with empty vector GFP or GFP-CtIP-WT. **E)** Cells were treated as in **Fig. 4D** and processed for DNA content analysis. Shown are the means of 2 to 4 independent experiments. **F) - I)** Immunoblots of cells treated with 10 μM KU-55933, 20 μM ETP-46464, 20 μM VE-821, or 1 μM NU7441 for 1 hour, then supplemented with 25 μM phleomycin (PHL) for 30 minutes **(F)** or 1 hour **(I)**, or treated with 82 J/m^2 of ultraviolet light (UV) and recovered for 1 hour in their presence **(G, H)**. **J)** Immunoblot of input and His PD fractions of HeLa His_{10}-SUMO-2 cells synchronized to mid-S phase and transfected with GFP-CtIP-WT or substitution mutants at residue T859. Shown is a representative result of 3 independent experiments. * indicates an exogenous CtIP immunoreactive band that is

not of interest; we speculate it is either lower molecular weight SUMO-2-modified CtIP, or unmodified tagged-CtIP retained in the His PD fraction due to overexpression.

Supplemental Figure S6: Related to Figure 4

A) U-2 OS cells were transfected with GFP-CtIP and either monomeric red fluorescent protein (RFP) empty vector or RFP-PCNA and processed for immunoprecipitation (IP) for the RFP tag. Prior to IP, a portion of lysate was saved as an input control. Shown is a representative result of at least 6 independent experiments. **B)** Chromatin fractionation experiment, with resulting soluble (S) or chromatin-enriched (P, pellet) fractions for U-2 OS cells stably expressing GFP-CtIP-WT, -K578R, -K896R, or -Δ515-518, or the parental U-2 OS cells. Histone H2A and α-tubulin serve as markers for the chromatin-enriched and soluble fractions, respectively.

Supplemental Figure S7: Related to Figure 6

A) Schematic diagrams of CtIP domain structure of FLAG-tagged wildtype (WT) CtIP and a panel of corresponding internal deletion mutants (D3 to D6); the regions deleted are denoted by the dotted line. Also indicated in the WT schematic are relevant phosphorylation and SUMOylation sites in this study. “Tet”: tetramerization domain; “dimeriz”: dimerization domain; “nuclease”: endonuclease domain, “PCNA interaction”: also known as PIP-Box. **B)** HeLa His_{10}-SUMO-2 cells were synchronized to mid-S phase. 24 hours before harvest, they were transfected with FLAG-CtIP-WT or the internal deletion mutants D3, D4, D5, and D6, then portioned into input control and His PD fractions and processed accordingly. Shown is a representative result of 2 independent experiments. * indicates a non-specific immunoreactive band. **C)** Asynchronous HeLa His_{10}-SUMO-2 cells were transfected with GFP empty vector or the indicated GFP-CtIP

constructs, portioned into input control and His PD fractions, and processed accordingly. **D)** Asynchronous HeLa His_{10}-SUMO-2 cells were transfected with the indicated HA-tagged CtIP constructs, portioned into input control and His pull-down (His PD) fractions, and processed accordingly. **E)** Clustal Omega (European Bioinformatics Institute, European Molecular Biology Laboratory) multiple sequence alignment was performed on the amino acid residue sequences of CtIP orthologues. The conserved lysine residue at K578 (in human CtIP) and the surrounding canonical SUMOylation motif are highlighted. * indicates complete identity; **:** indicates conservative substitutions; **-** indicates the residue is absent.

Supplemental Figure S8: Related to Figure 7

A) and B) Live cell time-lapse laser microirradiation experiments performed on U-2 OS cells depleted of endogenous CtIP with siRNA and transfected with siRNA-resistant GFP-CtIP-WT, -K578R, or -Δ515-518. **A)** shows images representative of the results from at least 3 independent experiments; white arrows indicate the linear region of laser microirradiation. **B)** displays the mean relative fluorescence intensity at the microirradiated region of 24 – 48 cells sourced from 3 independent experiments. **C)** HEK293 cells depleted of endogenous CtIP by siRNA were transiently transfected with GFP empty vector or GFP-CtIP-WT or -K578R, then immunoprecipitated (IP) for GFP. Prior to IP, a portion of lysate was saved as an input control. SDS-PAGE and immunoblotting were then performed on both input and IP fractions. The blots displayed are representative of at least 2 independent experiments. **D) and E)** Coomassie Brilliant Blue-stained SDS-PAGE gels showing purification of CtIP-WT- and -K578R or their phosphorylated versions (pCtIP) **(D)** or the MRN complex **(E)** from *Sf*9 cells. MW: molecular

weight standards. **F)** *In vitro* MRN endonuclease assay utilizing the products of **D)** and **E)** on a 5'-labeled streptavidin (S)-blocked 70 base pair (bp) double-stranded DNA substrate.

Supplemental Figure S9: K578 is Not Required for CtIP Recruitment During Replication Stress or its Interaction with PCNA

A) Stable cell lines expressing GFP-CtIP-WT, -K578R, or Δ515-518 were transfected with siRNA to CtIP and then treated or not with HU for 4 hours, then processed for IF staining. Left panel: representative images of the results from 4 independent experiments. Right panel: 826 to 1678 cells (for all except the untreated condition, these were γH2AX$^+$ cells) from 83 to 86 fields of view from 4 independent replicates were scored for the appearance of GFP-CtIP foci. **B)** U-2 OS cells were depleted of endogenous CtIP by siRNA and transfected with RFP-PCNA and either GFP empty vector or siRNA-resistant GFP-CtIP-WT or -K578R. A portion of lysate was saved as an input control, after which the remainder was processed for IP of the GFP tag. The result shown is representative of at least 4 independent experiments. **C)** U-2 OS cells stably expressing GFP-CtIP-WT or -K578R were depleted of endogenous CtIP by siRNA, then transfected with either RFP-PCNA or RFP empty vector and processed for IP of the RFP tag. Prior to IP, a portion of lysate was saved as an input control. The result shown is representative of at least 4 independent experiments. In the IP fraction, the RFP empty vector pulled down is 57.6% of the amount of RFP-PCNA-WT and -K578R pulled down. **D)** U-2 OS cells were co-transfected with RFP-PCNA and GFP-CtIP-WT, -K578R, or -Δ515-518 and processed for IP of the RFP tag. Prior to IP, a portion of lysate was saved as an input control. The result shown is representative of 2 independent experiments. **E)** U-2 OS cells stably expressing GFP-CtIP-WT or -K578R were depleted of endogenous CtIP by siRNA then treated for 4 hours with 2 or 8 mM HU or 0.8% H_2O (vehicle

control). U-2 OS cells without siRNA treatment and transfected with GFP empty vector served as a control sample. A portion of lysate was saved as an input control, after which the remainder was processed for IP of the GFP tag to detect co-immunoprecipitation of endogenous PCNA. The result shown is representative of 2 independent experiments.

Supplemental Figure S10: Related to Figure 8

A) Top panel: Labeling protocol for all DNA fiber experiments performed: cells were pulse-labeled with 5-chloro-2'-deoxyuridine (CldU) for 30 minutes, then with 5-iodo-2'-deoxyuridine (IdU) for 30 minutes, then treated with 2 mM HU for 4 hours to promote fork stalling. Bottom panel: a typical image of gravity-spread DNA fibers used for quantification of the IdU (labeled in red) / CldU (labeled in green) tract length ratio. **B)** IdU/CldU ratio scatterplot of spread DNA fibers from U-2 OS cells transfected with non-targeting siRNA (siCTRL) or siRNA directed to UBC9 (siUBC9) and treated according to **A)**. 210 to 222 fibers per condition were sourced from 2 independent experiments. **C)** HeLa His_{10}-SUMO-2 cells were transfected with the indicated GFP-CtIP constructs during synchronization to mid-S phase, then harvested. Cell pellets were portioned into input control and His PD fractions and processed accordingly. Shown is a representative result of at least 2 independent experiments. * indicates an exogenous CtIP immunoreactive band that is not of interest; we speculate it is either lower molecular weight SUMO-2-modified CtIP, or unmodified tagged-CtIP retained in the His PD fraction due to overexpression. **D)** Left panel: Coomassie Brilliant Blue-stained SDS-PAGE gel showing purification of DNA2 from *Sf*9 cells. Right panel: *in vitro* endonuclease assay utilizing DNA2 or phosphorylated (pCtIP) or not WT- or K578R-CtIP (**Fig. S8D**) on a 5'-labeled flap DNA substrate.

DNA2 activity was used as a positive control for nuclease activity. MW: molecular weight standards.

SUPPLEMENTAL TABLES

Supplemental Table S1: siRNAs Used in This Study

Target	Sense Sequence (5’-3’)	Manufacturer	Reference
CtIP	GCUAAAACAGGAACGAAUC	Millipore Sigma	(7)
PIAS1	GGAUCAUUCUAGAGCUUUA	Millipore Sigma	(8)
PIAS4	GGAGUAAGAGUGGACUGAA	Millipore Sigma	(8)
CBX4	GUACUACUACCAGCUCAACUU	Millipore Sigma	(7, 9)
UBC9	CAAAAAAUCCCGAUGGCAC	Millipore Sigma	(10)
Negative Control siRNA	N/A	Qiagen	#1022076

Supplemental Table S2: Primers for Site-Directed Mutagenesis

Mutant	Template	Source for Template	Primer Sequences (5’-3’)	Kit Used
GFP-CtIP-T859A	GFP-CtIP-WT	(7)	Forward:CTTTCCATACAAGTCTGAGCGGAAGGAAAACCAACTTCC<br>Reverse:GGAAGTTGGTTTTCCTTCCGCTCAGACTTGTATGGAAAG	QC
GFP-CtIP-T859E	GFP-CtIP-WT	(7)	Forward:AATATAACCTCTTTCCATACAAGTCTGCTCGGAAGGAAAACCAACTTCCCAAAAATT<br>Reverse:AATTTTTGGGAAGTTGGTTTTCCTTCCGAGCAGACTTGTATGGAAAGAGGTTATATT	QC
GFP-CtIP-Δ515-518	GFP-CtIP-WT	(7)	Forward:GTGACTCTTTATGAGGCTTTG<br>Reverse:GTTTTTAGAAGTCTCACTTCC	Q5
GFP-CtIP-K578R	GFP-CtIP-WT	(7)	Forward:GACAGCATTTTCTTCTCTTATTTGTAATGATGGTTTATTGTCTGGAGA<br>Reverse:TCTCCAGACAATAAACCATCATTACAAATAAGAGAAGAAAATGCTGTC	QC
HA-CtIP-K578R	pICE-HA-CtIP-siR-WT	Addgene #82030	Forward:ATTACAAATAAGAGAAGAAAATGCTG<br>Reverse:GATGGTTTATTGTCTGGAG	Q5
GFP-CtIP-K578R-K896R	GFP-CtIP-K896R	(7)	Forward:ATTACAAATAAGAGAAGAAAATGCTG<br>Reverse:GATGGTTTATTGTCTGGAG	Q5
GFP-CtIP-7KR-R578K	GFP-CtIP-7KR	(7)	Forward:TTACAAATAAAAGAAGAAAATGCTG<br>Reverse: TGATGGTTTATTGTCTGG	Q5
GFP-CtIP-N181A	GFP-CtIP-WT	(7)	Forward:AAGAAAGGAGGCCCCCCATGTCCGATAC<br>Reverse: CGTAGCCGGTTAACGCCA	Q5
GFP-CtIP-N181A-K578R	GFP-CtIP-K578R	generated for this report	Forward:AAGAAAGGAGGCCCCCCATGTCCGATAC<br>Reverse: CGTAGCCGGTTAACGCCA	Q5
GFP-CtIP-N289A-H290A	GFP-CtIP-WT	(7)	Forward:TCTGGAAGGAGCTGCCAAGAAACAGCCTTTTGAG<br>Reverse:CAGTGGTAGAGCTCATCAC	Q5
GFP-CtIP-N289A-H290A-K578R	GFP-CtIP-K578R	generated for this report	Forward:TCTGGAAGGAGCTGCCAAGAAACAGCCTTTTGAG<br>Reverse:CAGTGGTAGAGCTCATCAC	Q5

Primers were custom synthesized by Millipore Sigma.

QC: Quikchange II XL Site Directed Mutagenesis Kit (Agilent)
Q5: Q5 Site-Directed Mutagenesis Kit (New England Biolabs)

Supplemental Table S3: Primers for DNA Sequencing

Primer	Primer Sequence (5'-3')
CtIP 199 Forward	AGGGAACAGCAGAAAGTCCT
CtIP 692 Reverse	CTTTGGTCATAAGTGTCAGCTAC
CtIP 1523 Reverse	CCTTGGCTTTTCTCTTGACG
CtIP 1973 Forward	ATCCGGGAGCAGACCTTTC
CtIP 2289 Reverse	AGTGTGTAGTTTCTTTGTGGCA
CtIP 2409 Forward	GGAGAGAAGAAAACTGCTTGGG

Primers were custom synthesized by Millipore Sigma.

Supplemental Table S4: Primary Antibodies

Antigen	Host	Manufacturer	Catalogue Number	Application
β-Actin	rabbit	Millipore Sigma	A5060	IB (1:5000)
Phospho-ATM at S1981	mouse	Active Motif	39529	IB (1:2500)
BRCA1	rabbit	Bethyl Laboratories	A301-377A	IB (1:3000)
BrdU	mouse	GE Healthcare / Millipore Sigma	GERPN202	IF (1:750)
BrdU (for CldU)	rat	Abcam	ab6326	Fiber (1:100)
BrdU (for IdU)	mouse	BD Biosciences	347580	Fiber (1:100)
CBX4	rabbit	Atlas Antibodies / Millipore Sigma	HPA008228	IB (1:2000)
Phospho-Chk1 at S345	rabbit	Cell Signaling Technology	2348	IB (1:4000)
Phospho-Chk2 at T68	rabbit	Cell Signaling Technology	2197	IB (1:1000)
CtIP	mouse	Active Motif	61141	IB (1:16000)
CtIP	rabbit	Abcam	ab155988	IB (1:16000)
Cyclin A	rabbit	Santa Cruz Biotechnology	sc-751	IB (1:1000)
Cyclin D1	rabbit	Santa Cruz Biotechnology	sc-718	IB (1:1000)
Phospho-DNA-PKcs at S2056	rabbit	Abcam	ab18192	IB (1:3000)
EXO1	rabbit	Bethyl Laboratories	A302-640A	IB (1:2000)
FLAG tag	mouse	Sigma	F1804	IB (1:2000)
Geminin	rabbit	Cell Signaling Technology	5165	IB (1:1000)
GFP	mouse	Santa Cruz Biotechnology	sc-9996	IB (1:2000)
HA tag	mouse	BioLegend	901501	IB (1:1000)
His-probe	mouse	Santa Cruz Biotechnology	sc-8036	IB (1:500)
Histone H2A	rabbit	Upstate / Millipore Sigma	07-146	IB (1:2000)
Phospho-Histone H2AX at S139 (γH2AX)	rabbit	Active Motif	39118	IF (1:2000)
Phospho-Histone H2AX at S139 (γH2AX)	mouse	Millipore Sigma	05-636-I	IF (1:2000)
I-SceI	rabbit	Abcam	ab216263	IB (1:1000)
MRE11	mouse	GeneTex	GTX70212	IB (1:3000)
NBS1	rabbit	Novus Biologicals	NB100-143	IB (1:6000)<br>IF (1:500)
PCNA	mouse	Santa Cruz Biotechnology	sc-56	IB (1:2000)
PIAS1	rabbit	Abcam	ab32219	IB (1:1000)
PIAS4	rabbit	Abcam	ab58416	IB (1:3000)
Phospho-PP1α at T320	rabbit	Abcam	ab62334	IB (1:30000)
RAD50	mouse	Abcam	ab89	IB (1:4000)
RAD51	rabbit	Santa Cruz Biotechnology	sc8349	IF (1:200)
RanGAP-1	mouse	ThermoFisher Scientific	33-0800	IB (1:1000)
Phospho-Rpb1-CTD at S2	mouse	BioLegend	920204	IB (1:1000)

RFP	rabbit	Abcam	ab62341	IB (1:2000)
RPA2	mouse	Abcam	ab2175	IF (1:2000)
SUMO-2/3	mouse	Cytoskeleton	ASM23	IB (1:2500)
α-Tubulin	mouse	GeneTex	GTX628802	IB (1:10000)
α-Tubulin	mouse	Millipore Sigma	T6074	IB (1:10000)
α-Tubulin	mouse	Millipore Sigma	T6199	IB (1:5000)
UBC9	rabbit	Cell Signaling Technology	4786	IB (1:2000)

IB: immunoblot; IF: immunofluorescence; Fiber: DNA fiber assay

Supplemental Table S5: Secondary Antibodies

Antibody Conjugate	Manufacturer	Catalogue Number	Application
chicken anti-rat—Alexa Fluor 488	ThermoFisher Scientific	A-21470	Fiber (1:300)
goat anti-mouse—Alexa Fluor 546	ThermoFisher Scientific	A-21123	Fiber (1:300)
goat anti-mouse—HRP	LI-COR	926-80010	IB (1:5000)
goat anti-rabbit—HRP	LI-COR	926-80011	IB (1:5000)
donkey anti-mouse—IRDye 680RD	LI-COR	926-68072	IB (1:20000)
donkey anti-rabbit—IRDye 680RD	LI-COR	926-68073	IB (1:20000)
donkey anti-mouse—IRDye 800CW	LI-COR	926-32212	IB (1:20000)
donkey anti-rabbit—IRDye 800CW	LI-COR	926-32213	IB (1:20000)
donkey anti-mouse—Alexa Fluor 488	ThermoFisher Scientific	A-21202	IF (1:1000)
donkey anti-mouse—Alexa Fluor 594	ThermoFisher Scientific	A-21203	IF (1:1000)
donkey anti-rabbit—Alexa Fluor 594	ThermoFisher Scientific	A-21207	IF (1:1000)
donkey anti-rabbit—Alexa Fluor 647	ThermoFisher Scientific	A-31573	IF (1:300)
goat anti-mouse—Alexa Fluor 647	Abcam	ab150115	IF (1:300)

HRP: horseradish peroxidase; IB: immunoblot; IF: immunofluorescence; Fiber: DNA fiber assay

Supplemental Table S6: Extraction Buffers for Immunofluorescence Staining

Molecule	Buffer Composition	Conditions
GFP-CtIP, RAD51, RPA, Native BrdU	25 mM HEPES pH 7.9, 300 mM sucrose, 50 mM NaCl, 1 mM EDTA, 3 mM $MgCl_2$, 0.5% Triton X-100	ice-cold 2 times, 3 minutes each
MRE11-GFP	20 mM HEPES pH 7.5, 20 mM NaCl, 5 mM $MgCl_2$, 0.5% Nonidet P-40	ice-cold 2 times, 3 minutes each
NBS1	10 mM HEPES pH 7.0, 300 mM sucrose, 100 mM NaCl, 3 mM MgCl2, 0.7% Triton X-100, 0.3 mg/mL RNase A (added fresh)	room temperature 2 times, 5 minutes each

SUPPLEMENTAL REFERENCES

1. Pierce,A.J., Johnson,R.D., Thompson,L.H. and Jasin,M. (1999) XRCC3 promotes homology-directed repair of DNA damage in mammalian cells. *Genes Dev.*, **13**, 2633–2638.

2. Bowman,E.A. and Kelly,W.G. (2014) RNA polymerase II transcription elongation and Pol II CTD Ser2 phosphorylation: A tail of two kinases. *Nucleus*, **5**, 224–236.

3. Bach,S., Knockaert,M., Reinhardt,J., Lozach,O., Schmitt,S., Baratte,B., Koken,M., Coburn,S.P., Tang,L., Jiang,T., *et al.* (2005) Roscovitine targets, protein kinases and pyridoxal kinase. *J. Biol. Chem.*, **280**, 31208–31219.

4. Byth,K.F., Thomas,A., Hughes,G., Forder,C., McGregor,A., Geh,C., Oakes,S., Green,C., Walker,M., Newcombe,N., *et al.* (2009) AZD5438, a potent oral inhibitor of cyclin-dependent kinases 1, 2, and 9, leads to pharmacodynamic changes and potent antitumor effects in human tumor xenografts. *Mol. Cancer Ther.*, **8**, 1856–1866.

5. Dohadwala,M., da Cruz e Silva,E.F., Hall,F.L., Williams,R.T., Carbonaro-Hall,D.A., Nairn,A.C., Greengard,P. and Berndt,N. (1994) Phosphorylation and inactivation of protein phosphatase 1 by cyclin-dependent kinases. *Proc. Natl. Acad. Sci. U. S. A.*, **91**, 6408–6412.

6. Vassilev,L.T., Tovar,C., Chen,S., Knezevic,D., Zhao,X., Sun,H., Heimbrook,D.C. and Chen,L. (2006) Selective small-molecule inhibitor reveals critical mitotic functions of human CDK1. *Proc. Natl. Acad. Sci. U. S. A.*, **103**, 10660–10665.

7. Soria-Bretones,I., Cepeda-Garcia,C., Checa-Rodriguez,C., Heyer,V., Reina-San-Martin,B., Soutoglou,E., Huertas,P., Cepeda-García,C., Checa-Rodriguez,C., Heyer,V., *et al.* (2017) DNA end resection requires constitutive sumoylation of CtIP by CBX4. *Nat. Commun.*, **8**, 113.

8. Galanty,Y., Belotserkovskaya,R., Coates,J., Polo,S., Miller,K.M. and Jackson,S.P. (2009)

Mammalian SUMO E3-ligases PIAS1 and PIAS4 promote responses to DNA double-strand breaks. *Nature*, **462**, 935–939.

9. Sakamoto,Y., Watanabe,S., Ichimura,T., Kawasuji,M., Koseki,H., Baba,H. and Nakao,M. (2007) Overlapping roles of the methylated DNA-binding protein MBD1 and polycomb group proteins in transcriptional repression of HOXA genes and heterochromatin foci formation. *J. Biol. Chem.*, **282**, 16391–16400.

10. Lin,X., Liang,M., Liang,Y.-Y., Brunicardi,F.C. and Feng,X.-H. (2003) SUMO-1/Ubc9 promotes nuclear accumulation and metabolic stability of tumor suppressor Smad4. *J. Biol. Chem.*, **278**, 31043–31048.

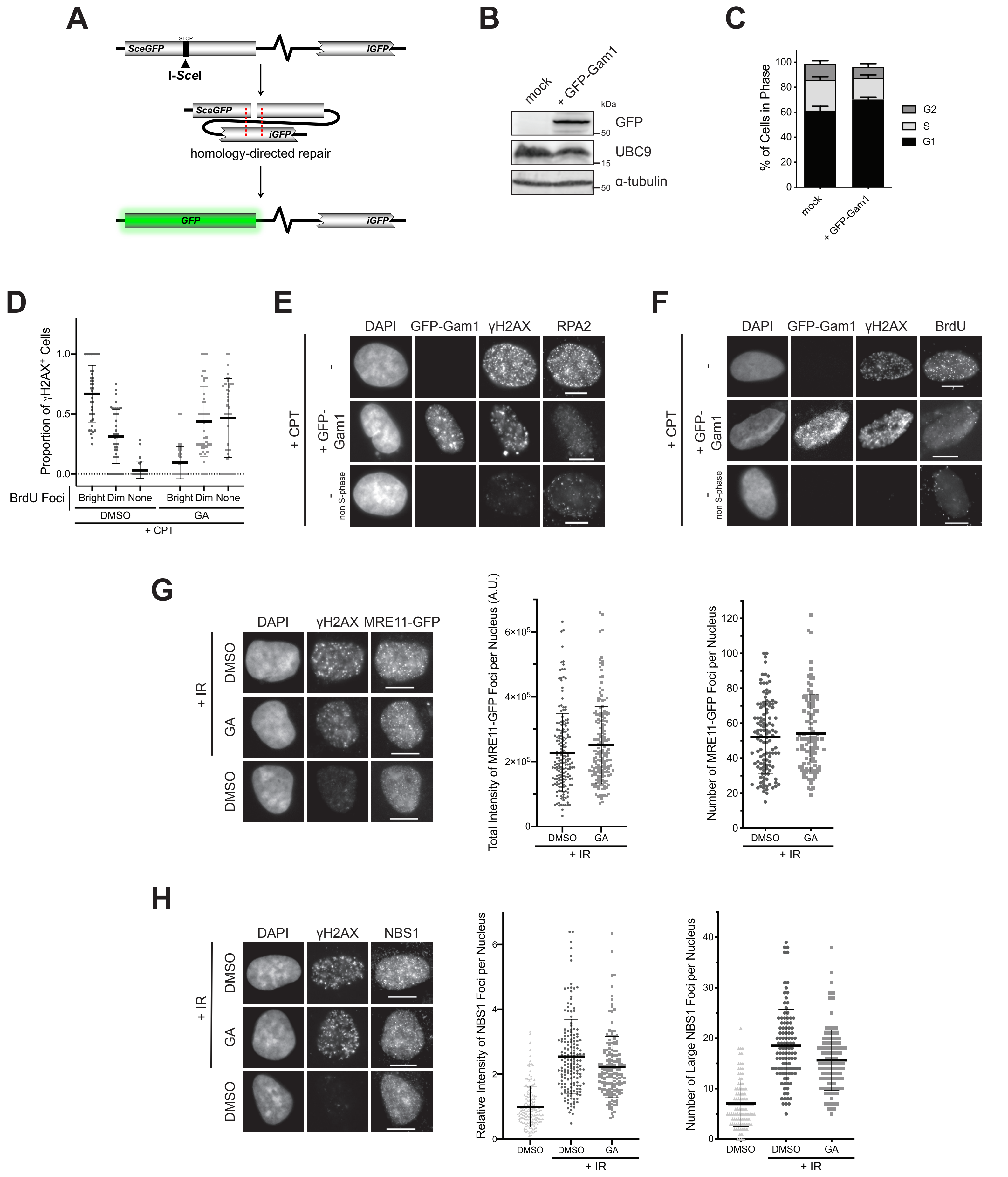
A
SceGFP
STOP
iGFP
I-SceI
SceGFP
iGFP
homology-directed repair
GFP
iGFP
B
mock
+ GFP-Gam1
kDa
GFP
50
UBC9
15
α-tubulin
50
C
% of Cells in Phase
G2
S
G1
mock
+ GFP-Gam1
D
Proportion of γH2AX+ Cells
BrdU Foci
Bright Dim None
Bright Dim None
DMSO
GA
+ CPT
E
DAPI GFP-Gam1 γH2AX RPA2
+ CPT
+ GFP-Gam1
non S-phase
F
DAPI GFP-Gam1 γH2AX BrdU
+ CPT
+ GFP-Gam1
non S-phase
G
DAPI γH2AX MRE11-GFP
+ IR
DMSO
GA
DMSO
Total Intensity of MRE11-GFP Foci per Nucleus (A.U.)
Number of MRE11-GFP Foci per Nucleus
DMSO GA
+ IR
H
DAPI γH2AX NBS1
+ IR
DMSO
GA
DMSO
Relative Intensity of NBS1 Foci per Nucleus
Number of Large NBS1 Foci per Nucleus
DMSO DMSO GA
+ IR

A

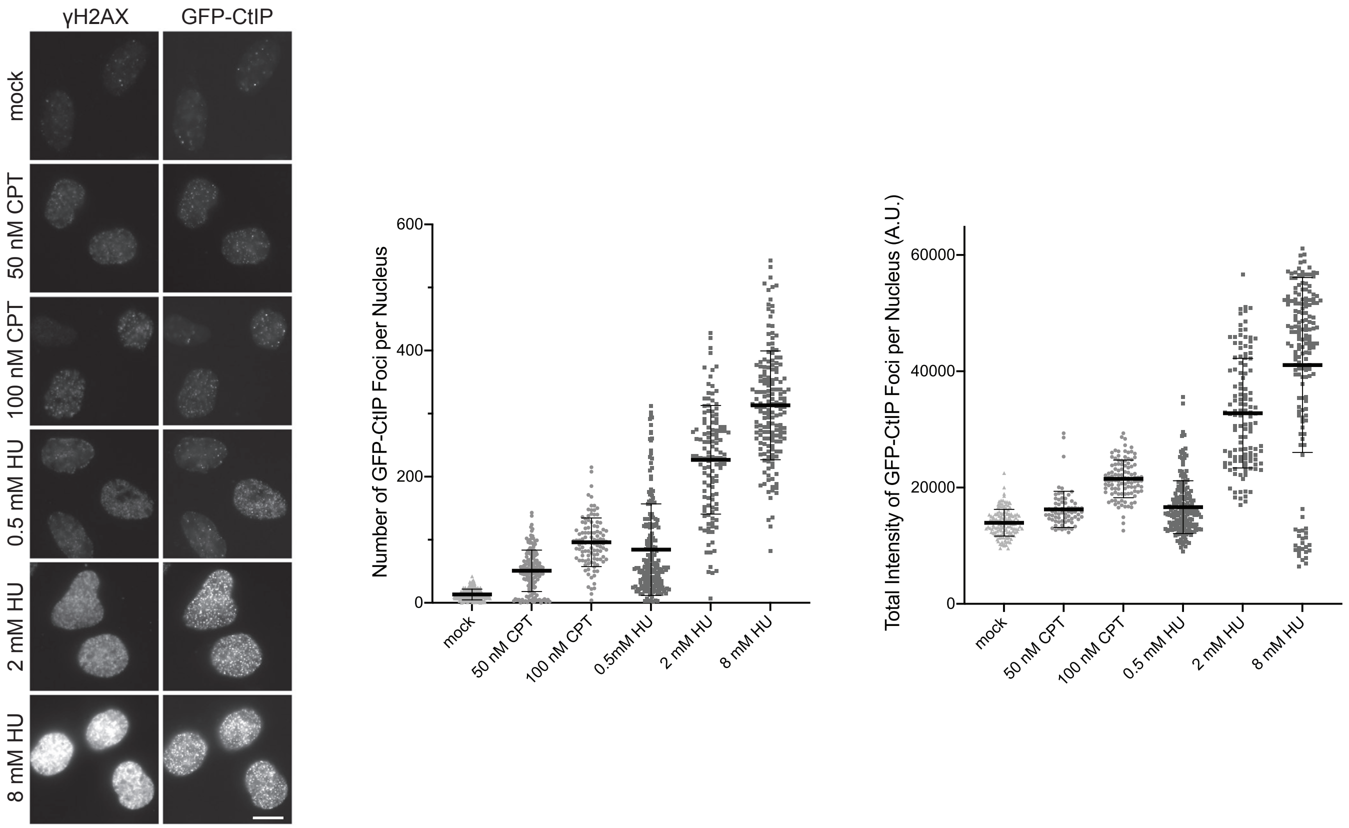
γH2AX
GFP-CtIP
mock
50 nM CPT
100 nM CPT
0.5 mM HU
2 mM HU
8 mM HU
Number of GFP-CtIP Foci per Nucleus
0
200
400
600
mock
50 nM CPT
100 nM CPT
0.5mM HU
2 mM HU
8 mM HU
Total Intensity of GFP-CtIP Foci per Nucleus (A.U.)
0
20000
40000
60000
mock
50 nM CPT
100 nM CPT
0.5 mM HU
2 mM HU
8 mM HU

B

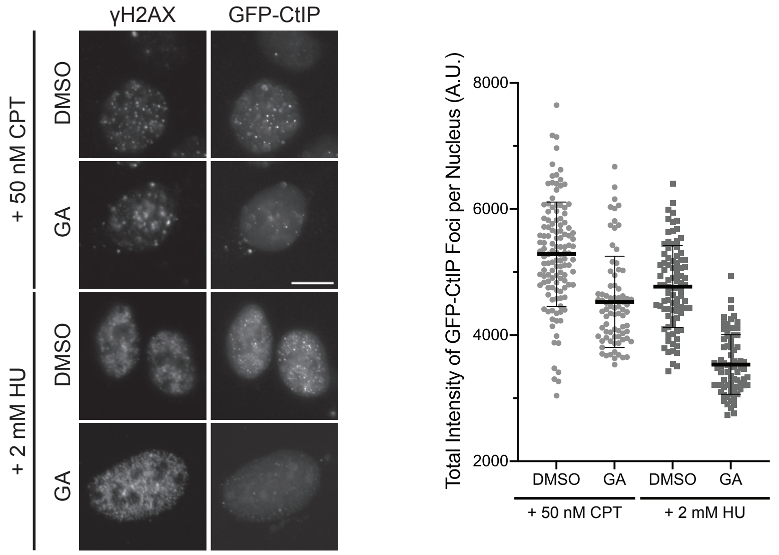
γH2AX
GFP-CtIP
+ 50 nM CPT
+ 2 mM HU
DMSO
GA
DMSO
GA
Total Intensity of GFP-CtIP Foci per Nucleus (A.U.)
2000
4000
6000
8000
DMSO
GA
DMSO
GA
+ 50 nM CPT
+ 2 mM HU

Locke *et al.* **Figure S3**

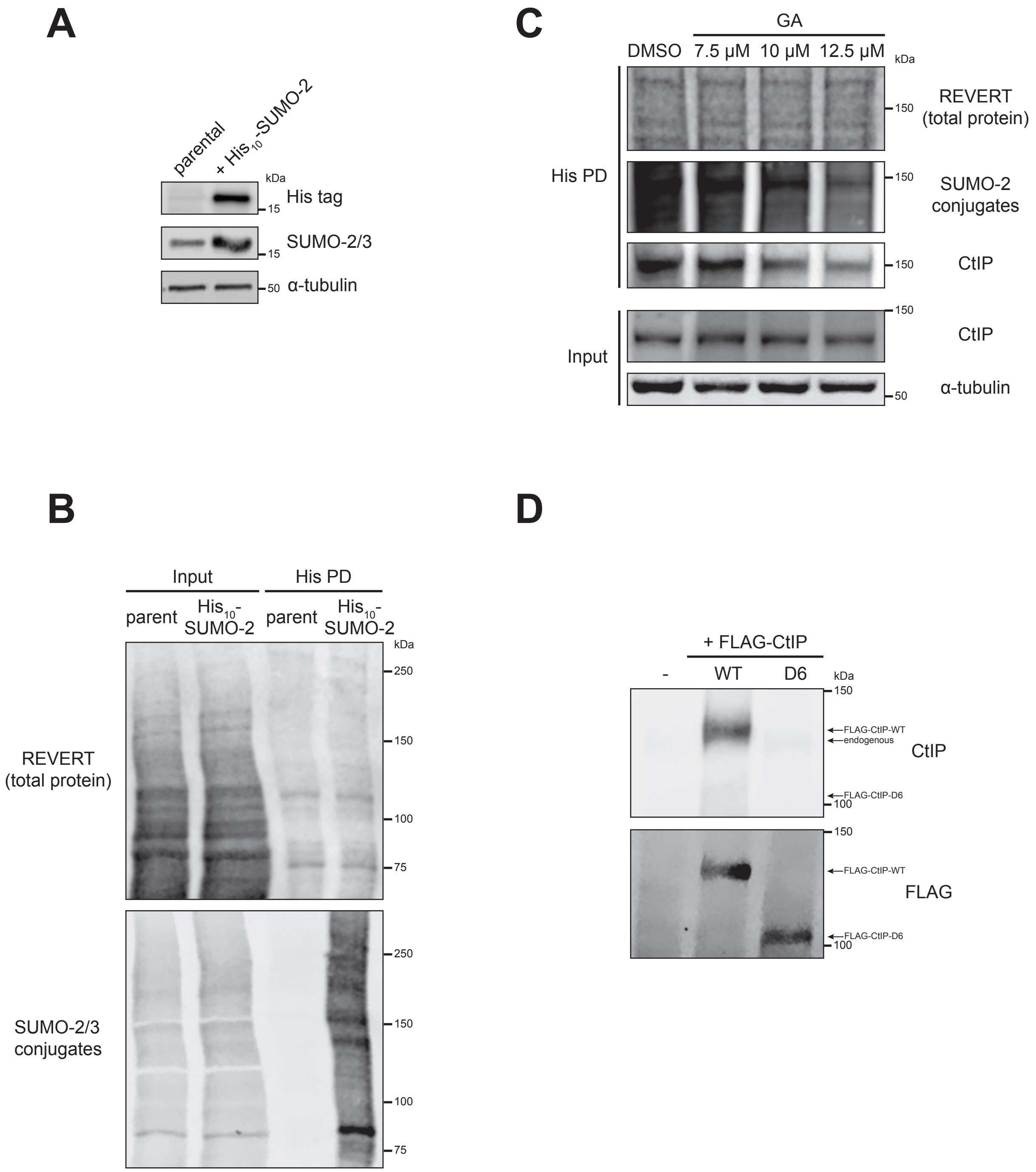

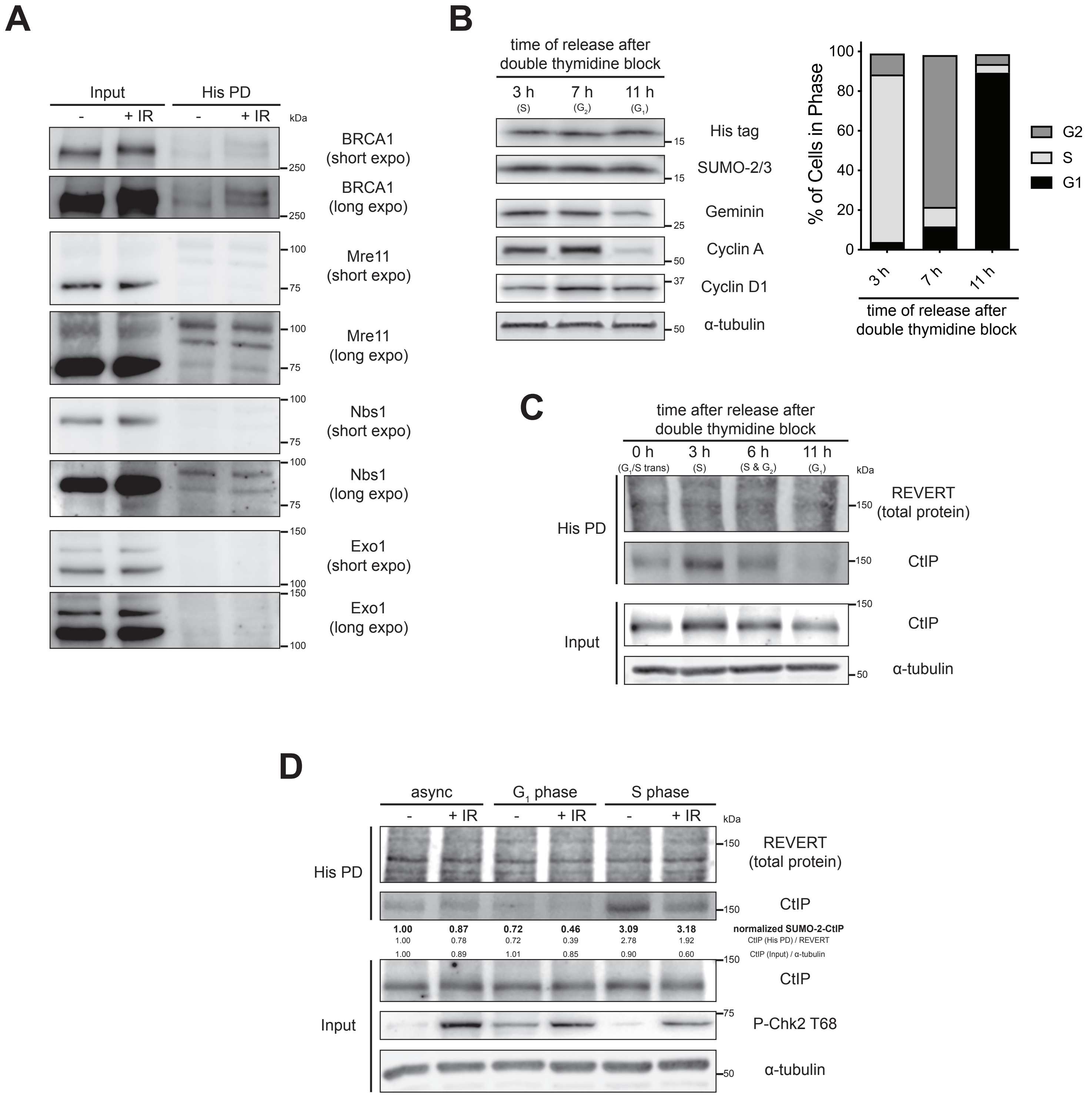
A
Input
His PD
-
+ IR
-
+ IR
kDa
BRCA1
(short expo)
BRCA1
(long expo)
Mre11
(short expo)
Mre11
(long expo)
Nbs1
(short expo)
Nbs1
(long expo)
Exo1
(short expo)
Exo1
(long expo)
250
100
75
150
B
time of release after
double thymidine block
3 h
(S)
7 h
(G2)
11 h
(G1)
His tag
SUMO-2/3
Geminin
Cyclin A
Cyclin D1
α-tubulin
15
25
50
37
% of Cells in Phase
0
20
40
60
80
100
G2
S
G1
3 h
7 h
11 h
time of release after
double thymidine block
C
time after release after
double thymidine block
0 h
(G1/S trans)
3 h
(S)
6 h
(S & G2)
11 h
(G1)
kDa
His PD
REVERT
(total protein)
CtIP
Input
CtIP
α-tubulin
150
50
D
async
G1 phase
S phase
-
+ IR
-
+ IR
-
+ IR
kDa
His PD
REVERT
(total protein)
CtIP
1.00
0.87
0.72
0.46
3.09
3.18
normalized SUMO-2-CtIP
1.00
0.78
0.72
0.39
2.78
1.92
CtIP (His PD) / REVERT
1.00
0.89
1.01
0.85
0.90
0.60
CtIP (Input) / α-tubulin
Input
CtIP
P-Chk2 T68
α-tubulin
150
75
50

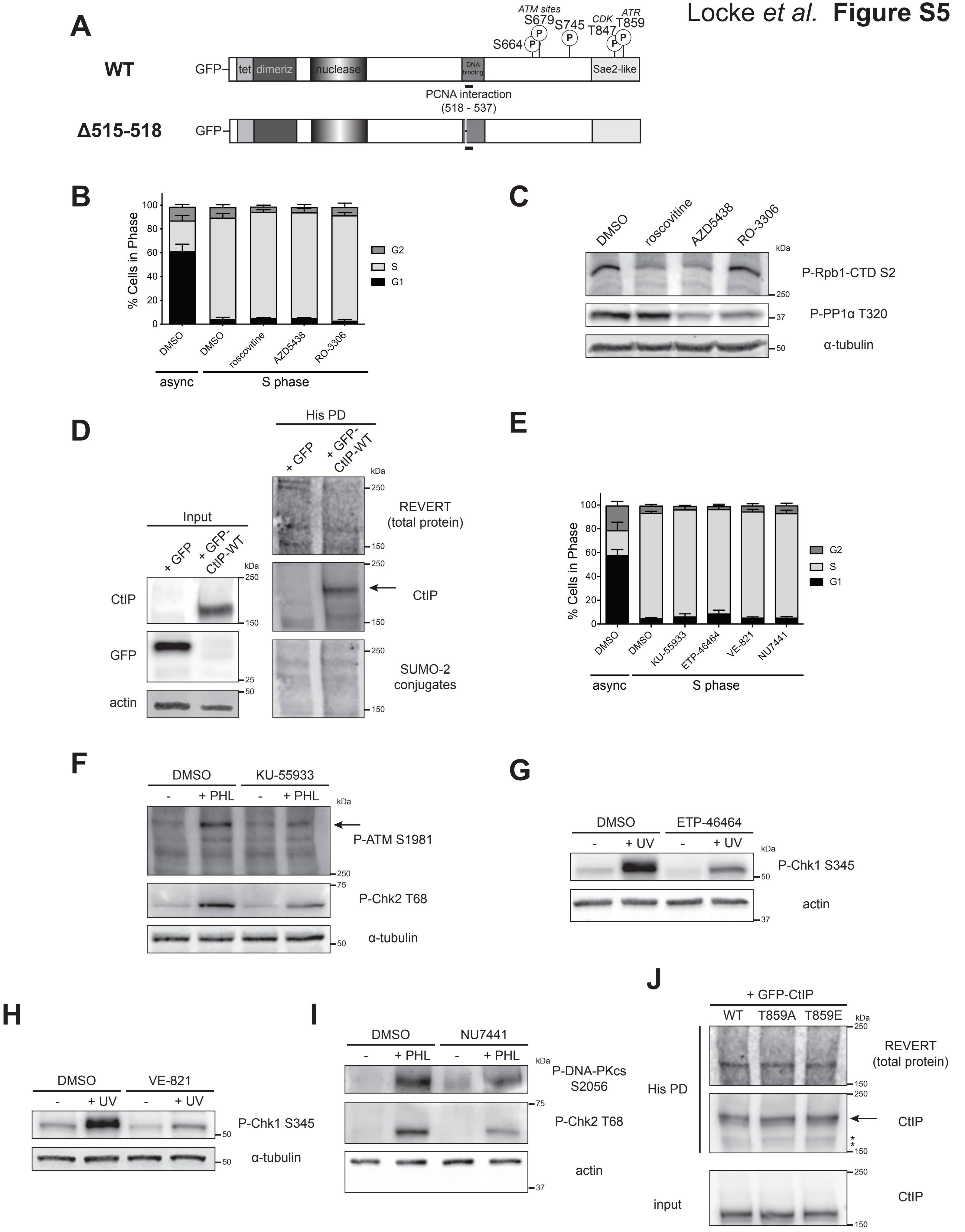
A
ATM sites
S679
S745
S664
CDK
T847
ATR
T859
P
WT
GFP
tet
dimeriz
nuclease
DNA binding
Sae2-like
PCNA interaction
(518 - 537)
Δ515-518
GFP
B
% Cells in Phase
G2
S
G1
DMSO
DMSO
roscovitine
AZD5438
RO-3306
async
S phase
C
DMSO
roscovitine
AZD5438
RO-3306
kDa
P-Rpb1-CTD S2
P-PP1α T320
α-tubulin
D
Input
+ GFP
+ GFP-CtIP-WT
kDa
CtIP
GFP
actin
His PD
REVERT
(total protein)
CtIP
SUMO-2
conjugates
E
% Cells in Phase
G2
S
G1
DMSO
DMSO
KU-55933
ETP-46464
VE-821
NU7441
async
S phase
F
DMSO
KU-55933
-
+ PHL
kDa
P-ATM S1981
P-Chk2 T68
α-tubulin
G
DMSO
ETP-46464
-
+ UV
kDa
P-Chk1 S345
actin
H
DMSO
VE-821
-
+ UV
P-Chk1 S345
α-tubulin
I
DMSO
NU7441
-
+ PHL
kDa
P-DNA-PKcs
S2056
P-Chk2 T68
actin
J
+ GFP-CtIP
WT
T859A
T859E
kDa
REVERT
(total protein)
His PD
CtIP
input
CtIP

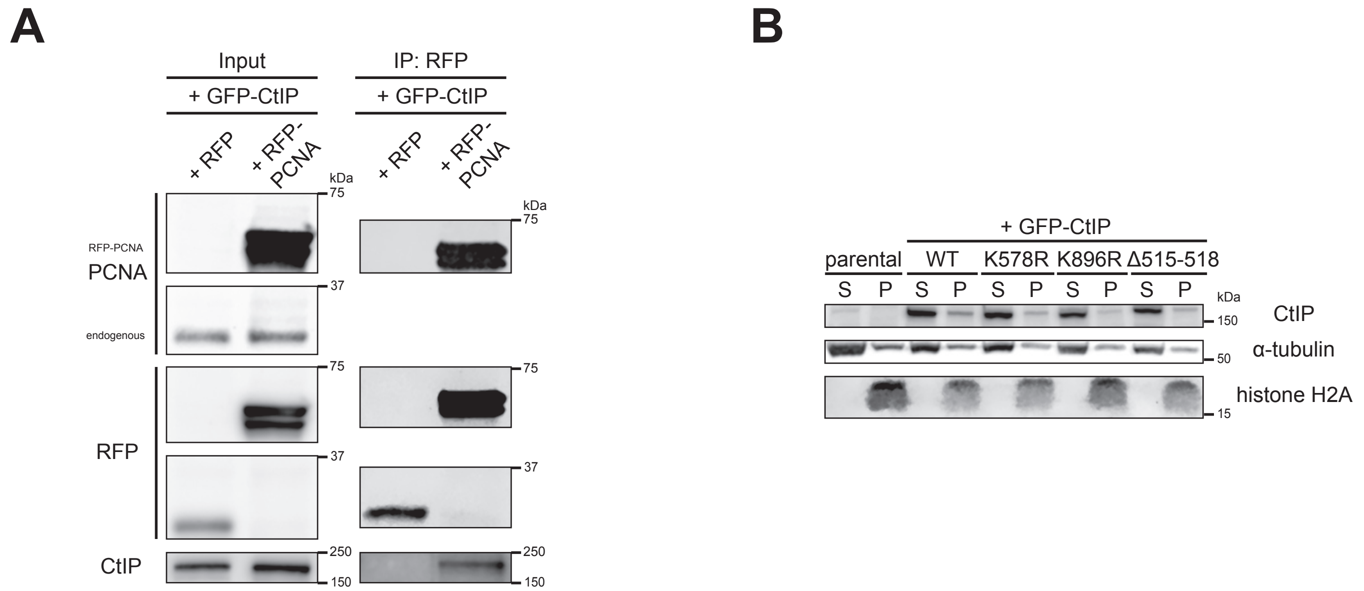
A
Input
+ GFP-CtIP
IP: RFP
+ GFP-CtIP
+ RFP
+ RFP-PCNA
kDa
75
37
250
150
RFP-PCNA
PCNA
endogenous
RFP
CtIP
B
+ GFP-CtIP
parental
WT
K578R
K896R
Δ515-518
S
P
CtIP
α-tubulin
histone H2A
50
15

Locke *et al.* **Figure S7**

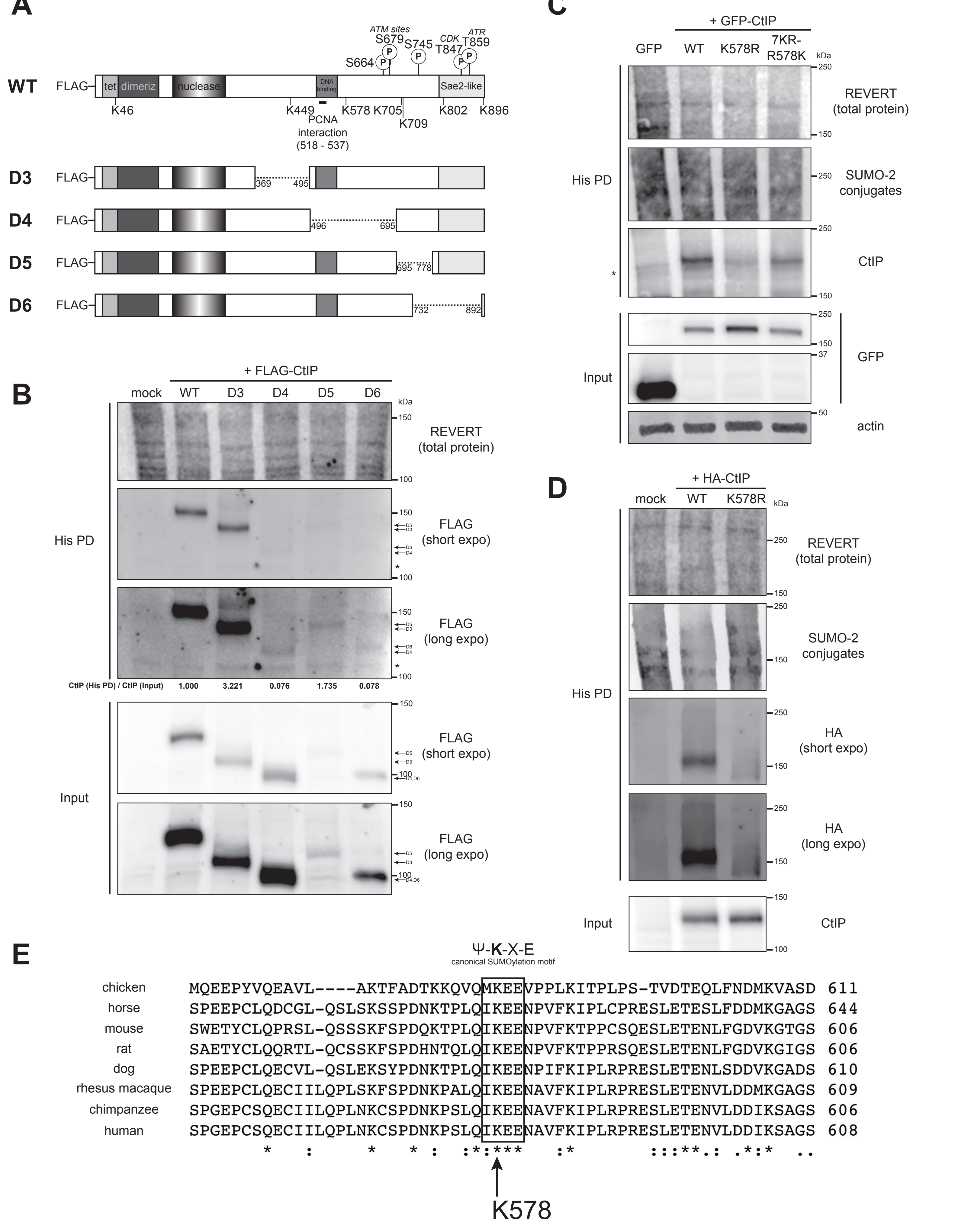

Locke *et al.* **Figure S8**

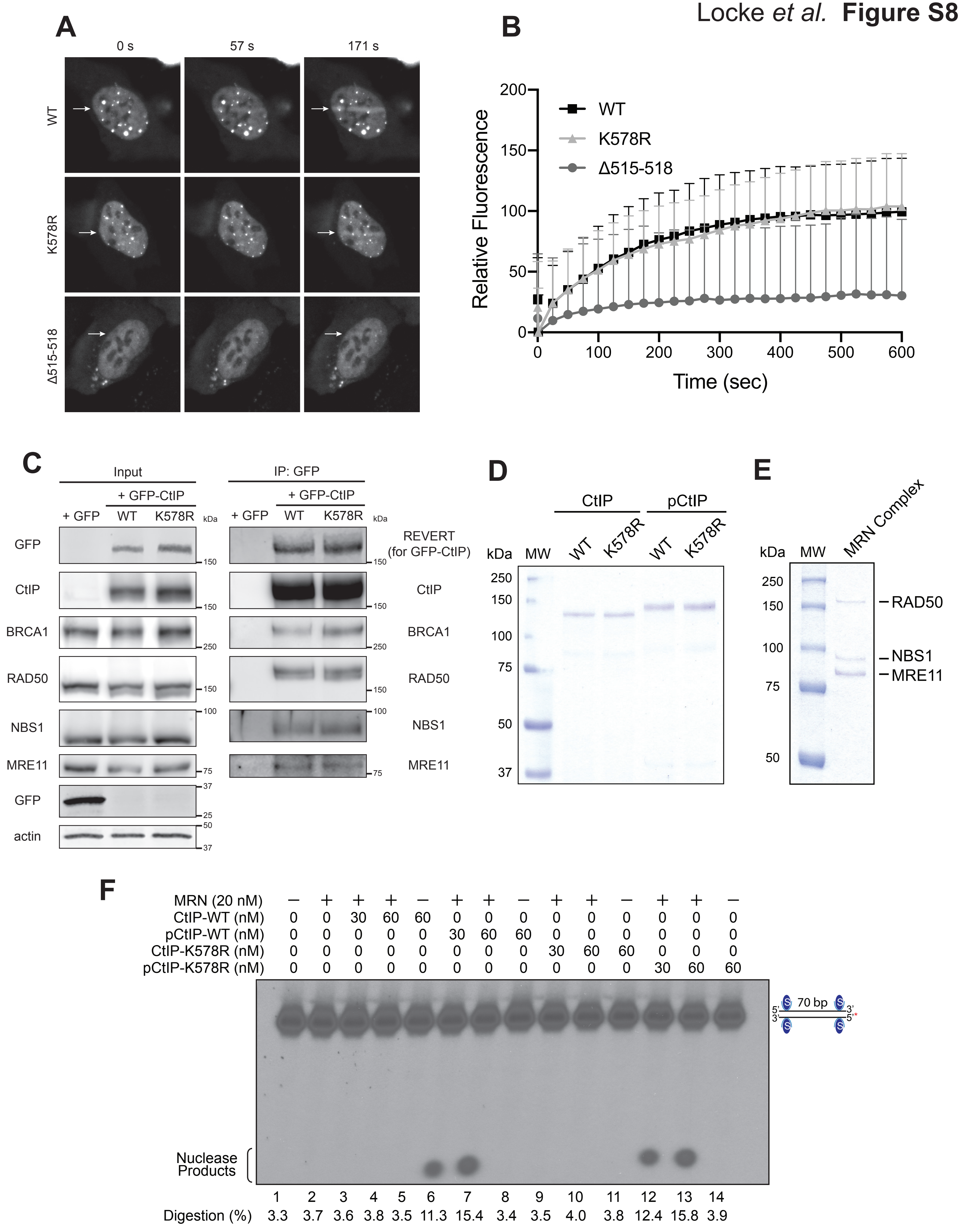

Locke *et al.* **Figure S9**

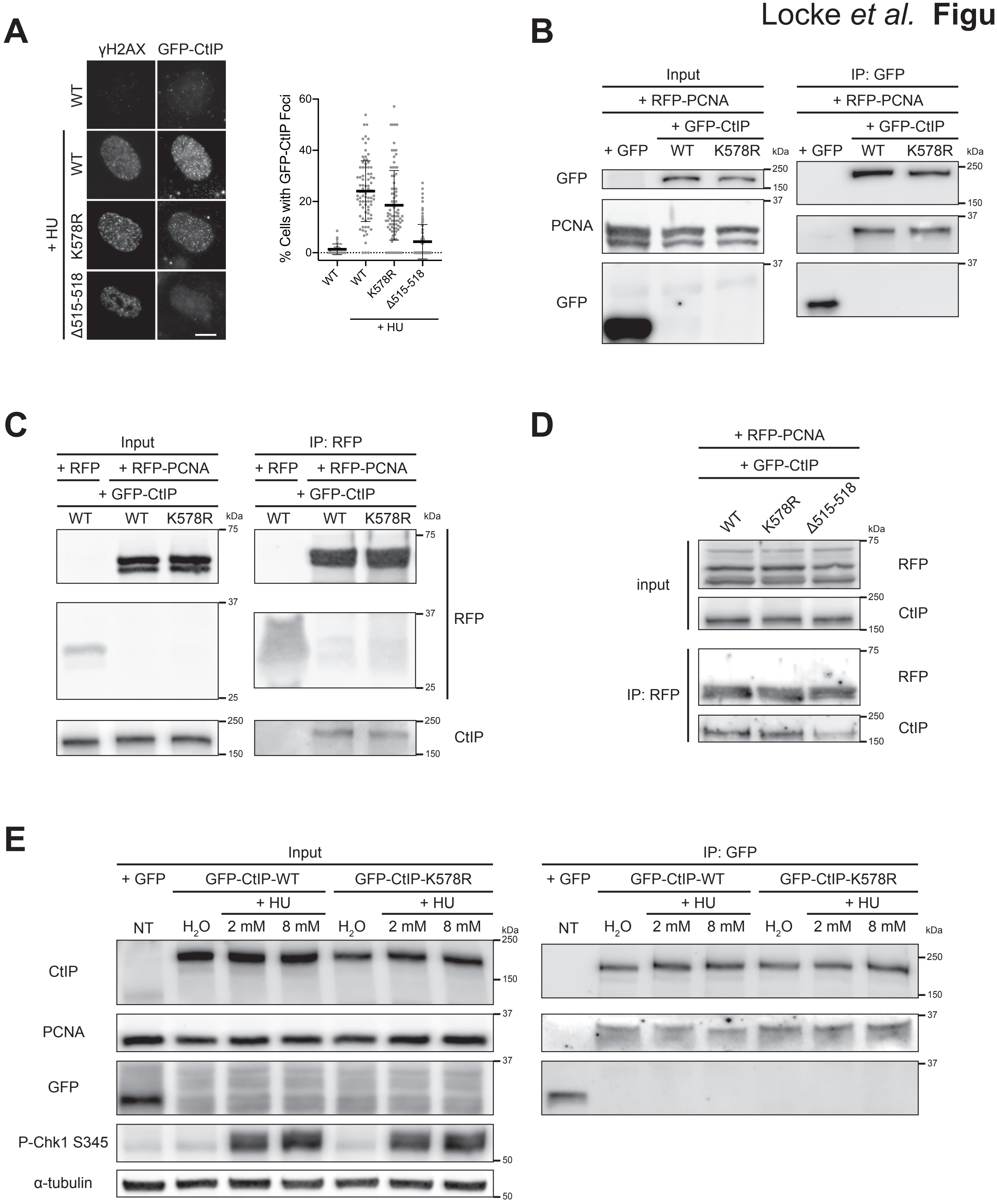

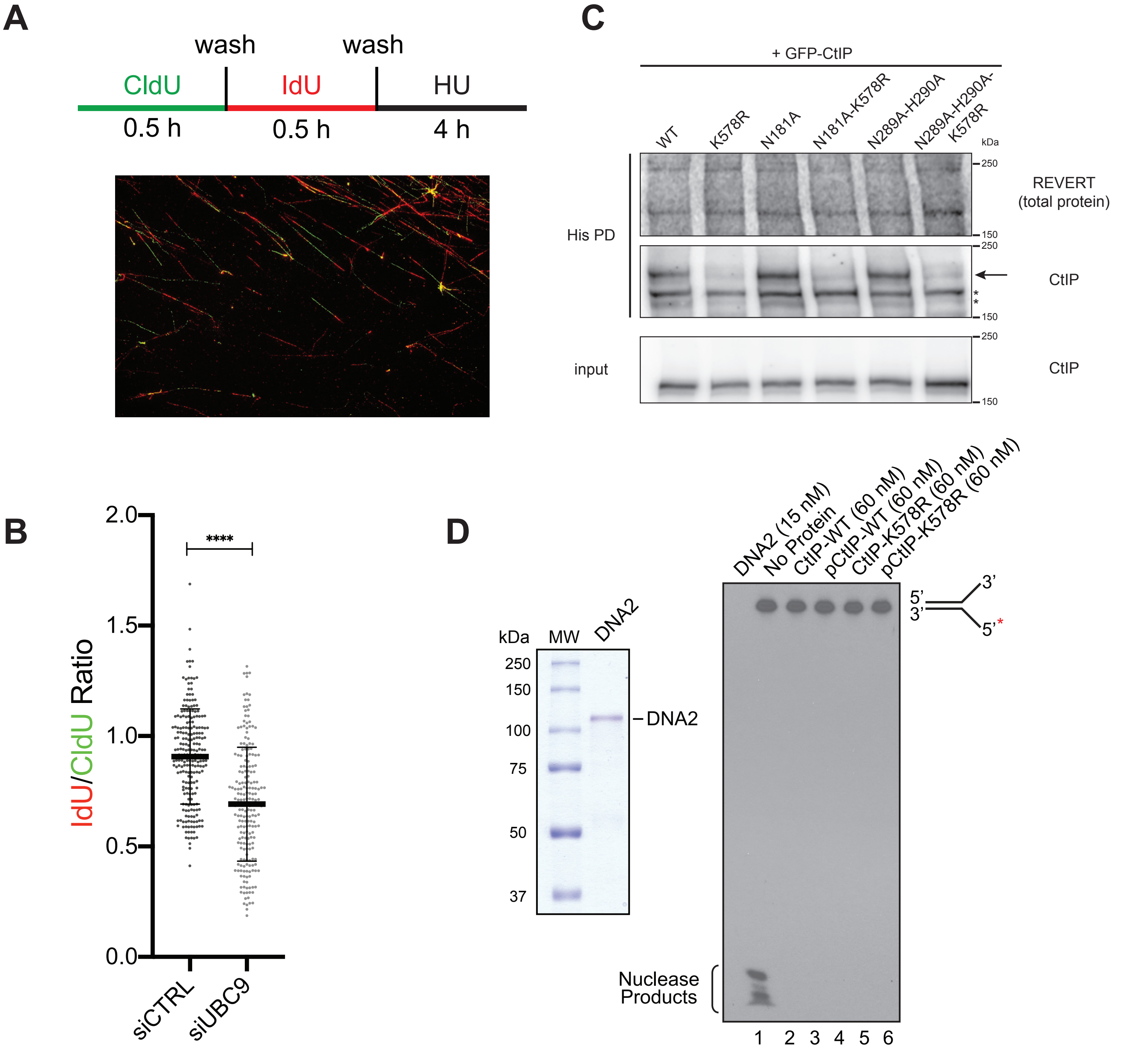
A
wash
wash
CldU
IdU
HU
0.5 h
0.5 h
4 h
B
IdU/CldU Ratio
2.0
1.5
1.0
0.5
0.0
****
siCTRL
siUBC9
C
+ GFP-CtIP
WT
K578R
N181A
N181A-K578R
N289A-H290A
N289A-H290A-K578R
kDa
250
150
REVERT (total protein)
His PD
CtIP
input
CtIP
D
kDa
MW
DNA2
250
150
100
75
50
37
DNA2
DNA2 (15 nM)
No Protein
CtIP-WT (60 nM)
pCtIP-WT (60 nM)
CtIP-K578R (60 nM)
pCtIP-K578R (60 nM)
5'
3'
3'
5'*
Nuclease Products
1 2 3 4 5 6
